# Multi-functional bionic nanoparticles for precise neuronal targeting and synergistic therapy in Alzheimer's disease

**DOI:** 10.1016/j.mtbio.2026.103617

**Published:** 2026-08-28

**Authors:** Yue Na, Chi Liu, Xinming Bi, Shumeng Liu, Yue Xing, Bohan Zhang, Tongfeng Liu, Ning Zhang, Fang Geng

**Affiliations:** a College of Life Science and Technology, Harbin Normal University, Harbin, Heilongjiang, 150025, PR China; b Key Laboratory of Photochemistry Biomaterials & Energy Storage Materials of Heilongjiang Province, College of Chemistry & Chemical Engineering, Harbin Normal University, Harbin, Heilongjiang, 150025, PR China; c College of Pharmacy, Heilongjiang University of Chinese Medicine, Harbin, Heilongjiang, 150004, PR China

**Keywords:** Alzheimer's disease, Synergistic therapy, Neuronal targeting, Multi-functional bionic nanoparticles

## Abstract

Alzheimer's disease (AD) is characterized by core pathological mechanisms including amyloid-β plaque aggregation, cholinergic dysfunction, and neuronal apoptosis. Given the complex interplay of these pathways, combination therapies are emerging as more effective strategies than single-target approaches. The success of such therapies depends on rational target selection and optimal regimen design. Herein, we developed a multifunctional biomimetic nanocarrier, B6/Tet1-RBCm-NPs (BTRN), for precise neuron-targeted combination therapy. The nanoparticles (NPs) core was fabricated using safe, biocompatible poly (lactic-co-glycolic acid) (PLGA) to co-deliver natural product genistein and mature marketed donepezil, which act synergistically to enhance therapeutic efficacy. The core was further modified with endogenous red blood cell membranes, providing effective immune evasion and a prolonged circulation lifetime. Functionalization with the blood-brain barrier (BBB)-penetrating peptide B6 and the neuron-targeting peptide Tet1 confers excellent BBB penetration and specific accumulation in diseased neurons. Collectively, BTRN treats AD through multiple mechanisms, including reducing Aβ production, regulating cholinergic function, and inhibiting neuronal apoptosis. This biomimetic system presents a promising therapeutic strategy and offers valuable insights for the clinical translation of combination therapies in AD.

## Highlights

• BTRN has excellent BBB penetration and neuron-targeting ability.
• Endogenous RBC membranes provide BTRN with immune evasion and long circulation.
• Safe, non-toxic PLGA carrier enables BTRN's clinical translation research.
• Genistein and donepezil combine synergistically, enhancing each other's effects.
• BTRN treats AD via Aβ reduction, cholinergic regulation, and apoptosis inhibition.

## Graphical abstract

**Figure:**
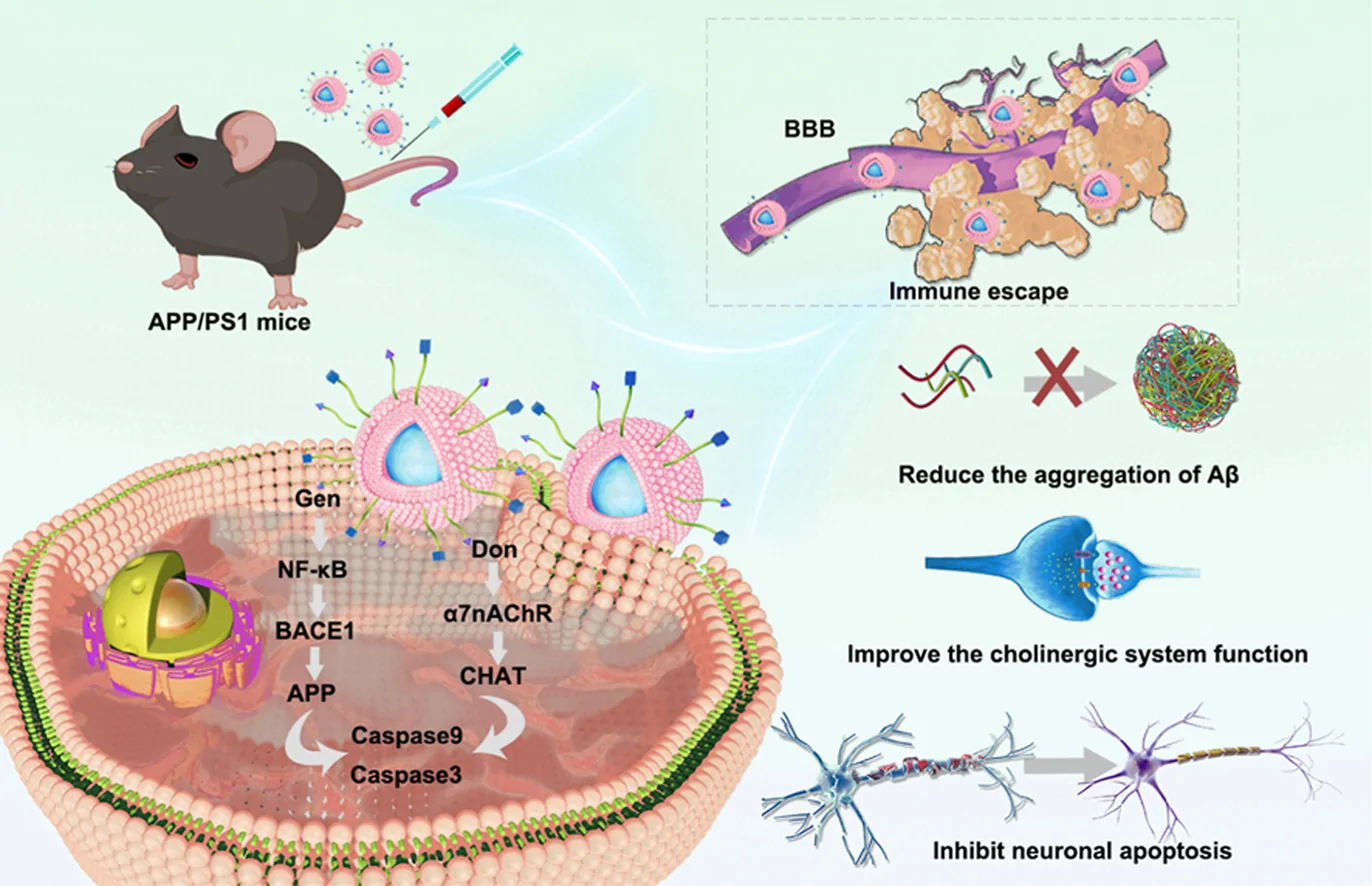

## 1 Introduction

Alzheimer's disease (AD) is a progressive neurodegenerative disorder with a complex pathogenesis [1]. The common pathological mechanisms of AD include Aβ aggregation, cholinergic system dysfunction, and neuronal apoptosis [[2], [3], [4]]. Aβ aggregation and cholinergic system dysfunction can lead to insufficient cellular energy supply, thereby causing neuronal apoptosis and loss [5,6]. Currently, a series of drugs and vaccines based on the “amyloid hypothesis” and “cholinergic system dysfunction” have been designed [7,8]. Despite the conditional FDA approval of the anti-amyloid monoclonal antibody aducanumab, most Aβ-targeted therapies have proven unsuccessful in clinical trials [9]. Similarly, while the cholinesterase inhibitor donepezil remains a clinical mainstay, it is associated with significant peripheral side effects [10]. This therapeutic limitation largely stems from the multifactorial nature of Alzheimer's disease, wherein complex interactions among multiple pathophysiological pathways undermine therapies that target only a single factor [11]. In recent years, combination therapy has been considered the most promising approach to overcome the complexity of mechanisms in AD treatment [[12], [13], [14]].

Aβ-mediated neuronal apoptosis is a core pathological driver of AD, contributing to irreversible neuronal depletion and the progressive cognitive decline characteristic of the disorder [15]. Genistein (Gen), one of the most biologically active natural flavonoids, has emerged as a promising neuroprotective agent for AD, with extensive research confirming its capacity to effectively mitigate Aβ-induced neuronal apoptosis in both *in vitro* and *in vivo* AD models [16]. Gen reduces pathological Aβ production through targeting the nuclear factor-κB (NF-κB)/β-site amyloid precursor protein cleaving enzyme 1 (BACE1)/amyloid precursor protein (APP) signaling pathway, via this pathway it suppresses NF-κB activation, downregulates the key Aβ-generating enzyme BACE1, limits the pathological proteolysis of APP into toxic Aβ fragments, and consequently alleviates Aβ neurotoxicity and improves AD-related pathological and cognitive manifestations [17]. However, Gen has only a limited effect on cognitive recovery and memory improvement *in vivo* attribute to its poor water solubility, low bioavailability, poor brain accumulation, and lack of neuronal targeting [18].

The depletion of brain ACh, a key neurotransmitter governing learning, memory, and synaptic plasticity, is a central driver of these cholinergic system deficits in AD [19]. Donepezil (Don), a clinically validated reversible non-competitive AChE inhibitor, is thus indispensable for AD therapy: it exerts its therapeutic effects by blocking AChE-mediated hydrolysis of ACh, thereby elevating central ACh levels, restoring cholinergic neurotransmission, and ameliorating the cognitive impairments associated with AD [20]. Despite its clinical utility, Don suffers from poor targeting specificity; this limitation restricts its accumulation at the intended central nervous system (CNS) sites of action and leads to off-target inhibition of peripheral AChE, culminating in a range of adverse effects (e.g., gastrointestinal disturbances and bradycardia) that compromise patient compliance [21]. Against this backdrop, the construction of a targeted delivery platform with robust BBB-crossing efficiency is critical to facilitating the synergistic co-administration of Gen and Don for AD treatment.

Nanoparticle-based drug delivery systems can address the shortages in AD treatment and provide promising solutions for neurodegenerative diseases such as AD [[22], [23], [24]]. However, developing effective drug delivery system for AD remains a challenge. On the one hand, the blood-brain barrier (BBB), as a homeostatic defense barrier of the brain, restricts the entry of almost all macromolecular drugs and over 98% of small molecular drugs [25,26]. On the other hand, even following successful BBB penetration, the intricate brain microenvironment hinders the specific delivery of therapeutics to neurons or AD lesions [27,28]. Therefore, it is thus critical to engineer a nano-drug delivery system that combines both precise targeting and efficient delivery capabilities [[29], [30], [31]].

Overcoming the BBB remains a major bottleneck for CNS drug delivery, and receptor-mediated transcytosis (RMT) has been validated as one of the most practical and effective pathways to address this challenge [32,33]. Notably, the transferrin receptor (TfR), a well-characterized RMT receptor, is abundantly expressed on the luminal surface of brain microvascular endothelial cells (BMECs), the core component of the BBB, as well as on choroid plexus epithelial cells, neurons, and astrocytes in the brain parenchyma [34]. For nanoparticle-based delivery systems, surface modification with the B6 peptide (CGHKAKGPRK) enables targeted engagement with transferrin, which forms a natural complex with TfR on BMECs. This specific interaction initiates TfR-dependent endocytosis and subsequent transcytosis across BMECs, ultimately shuttling the B6-decorated NPs across the BBB to reach the CNS [35].

Beyond BBB penetration, achieving precise neuronal targeting within the CNS is equally critical for AD therapy, and Tet1, a 12-amino-acid peptide (HLNILSTLWKYR), selectively binds to sphingomyelin and ganglioside GT1B, lipid components highly expressed on neuronal membranes, thereby enabling neuron-specific targeting [36]. In addition to peptide-mediated targeting, biomimetic membrane camouflage has emerged as a strategy to enhance *in vivo* delivery performance; erythrocytes are the most abundant anucleate blood cells in mammals and are the main medium for oxygen transport. Their cell membranes can be readily isolated and purified [37]. Moreover, the self-identification molecule CD47 on the surface of erythrocytes endows encapsulated NPs with a longer circulation half-life and immune evasion function [38]. Thus, by camouflaging NPs with an erythrocyte membrane, the system is anticipated to achieve systemic stability and immune evasion [39]. Crucially, this protective effect synergizes with the membrane-anchored B6 and Tet1 peptides, which are incorporated via lipid insertion, to promote targeted delivery across the BBB and to brain neurons.

**Fig. 1:**
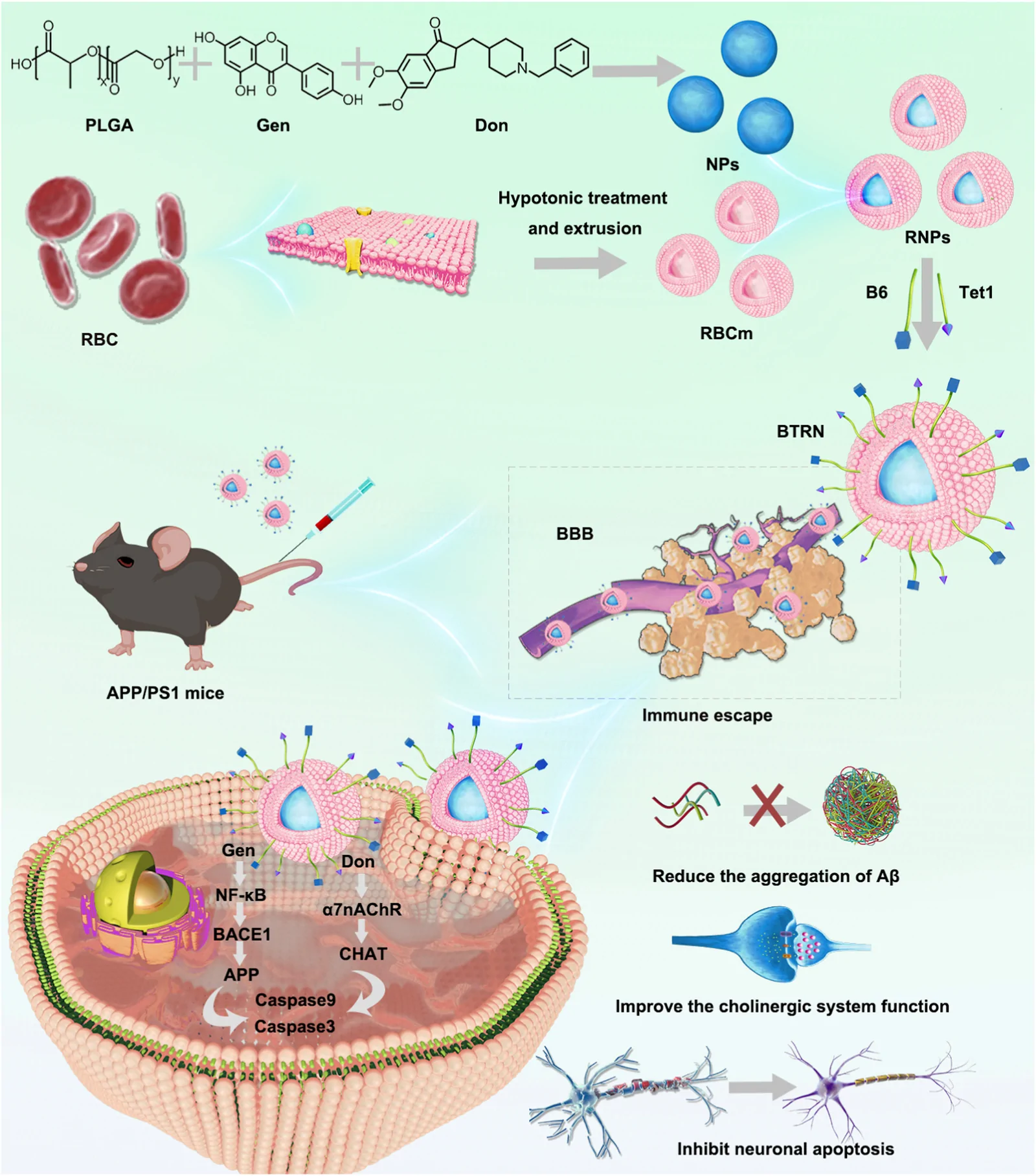
**Schematic illustration and mechanism of action of BTRN**. BTRN crosses the BBB, targets neuronal cells, and exerts therapeutic effects for AD by reducing Aβ aggregation, improving cholinergic function, and inhibiting neuronal apoptosis.

In this study, a multifunctional biomimetic nano delivery system (BTRN) was developed via a three-step assembly process: co-encapsulation of Gen and Don into a PLGA core, coating of the core with a natural erythrocyte membrane, and surface modification with B6 and Tet1 targeting peptides (Fig. 1). BTRN penetrates the BBB under the action of B6 and targets neurons under the action of Tet1 through self-erythrocyte membrane camouflage, allowing the effective drugs to be released in neurons. Gen reduces the generation of Aβ through the NF-κB/BACE1/APP pathway, while Don enhances cholinergic neurotransmission by regulating the level of acetylcholine in the brain. These mechanisms synergistically reduce neuronal apoptosis and thereby improve the cognitive function of APP/PS1 mice. In essence, BTRN is designed to simultaneously mitigate Aβ pathology, restore cholinergic neurotransmission, and prevent neuronal loss, thereby presenting a potential therapeutic approach for AD.

## 2 Materials and methods

### 2.1 Materials

PLGA was purchased from Daigang Bioengineering Co., Ltd. (Shandong, China). Genistein and Donepezil were obtained from Shanghai Yuanye Biotechnology Co., Ltd. (Shanghai, China). FITC was sourced from Beijing Saierui Biotechnology Co., Ltd. (Beijing, China). DiR (a near-infrared membrane probe) was procured from Beijing Innokon Biotechnology Co., Ltd. (Beijing, China). DSPE-PEG_2000_-Mal was acquired from Chongqing Yusai Pharmaceutical Technology Co., Ltd. (Chongqing, China). B6, Tet1, and Aβ_25-35_ were ordered from Shanghai Qiangyao Biotechnology Co., Ltd. (Shanghai, China). The BCA protein assay kit and TUNEL cell apoptosis detection kit was purchased from Beyotime Biotechnology Co., Ltd. (Shanghai, China). Antibodies against CD47 was obtained from Abcam (Shanghai, China), BACE1, NF-κB were obtained from Bioss (Beijing, China), SIRT1, APP, Aβ, ChAT, α7-nAchR were obtained from CST (Shanghai, China), Bax, Bcl-2, cleaved-caspase 3, cleaved-caspase 9, and NeuN were obtained from Proteintech (Wuhan, China). All other reagents were of analytical grade and purchased from local suppliers.

### 2.2 Cell culture and animal experimentation

The HT22 cell line was purchased from iCell Bioscience Inc. (Shanghai, China). The hCMEC/D3, U-118MG, and RAW 264.7 cell lines were obtained from Fenghui Biotechnology Co., Ltd. (Hunan, China). HT22, U-118MG, and RAW 264.7 cells were cultured in DMEM medium supplemented with 10% fetal bovine serum (FBS) and 1% penicillin-streptomycin. hCMEC/D3 cells were cultured in EGM-2 medium. All cells were maintained at 37°C in a humidified environment containing 5% CO_2_.

C57BL/6J mice and APP/PS1 mice were provided by GemPharmatech Co., Ltd. (Jiangsu, China). All animal experiments were conducted in compliance with the ethical guidelines for animal research established by the Animal Experiment Management Committee of Heilongjiang University of Chinese Medicine and were approved by the Animal Ethics Committee (2024090601).

### 2.3 Preparation of NPs

Firstly, we prepared the core of NPs loaded with genistein and donepezil by the emulsion solvent evaporation method. Specifically, genistein (1 mg), donepezil (1 mg), and PLGA (20 mg) were dissolved in organic solvents as the oil phase, and deionized water containing 1% PVA was used as the aqueous phase. The oil phase was slowly added to the aqueous phase under stirring, and the mixture was ultrasonicated in an ice bath to form an emulsion. The organic solvents were removed by rotary evaporation, and the NPs were repeatedly centrifuged and washed with deionized water to remove the surfactants, thus obtaining the nanoparticle core.

### 2.4 Preparation of RNPs

Rat whole blood was placed in a heparin sodium tube and centrifuged to obtain red blood cells. The red blood cells were mixed with Tris-HCl buffer at a volume ratio of 1:40 and hemolyzed at 4°C for 2 h. The hemoglobin was removed by centrifugation to obtain red blood cell membranes (RBCm), which were stored at 4°C. The RBCm were repeatedly extruded with NPs (Gen/Don) using a liposome extruder to obtain RBCm-NPs (Gen/Don) (RNPs).

### 2.5 Preparation of BTRN

DSPE-PEG_2000_-Mal was reacted with B6 and Tet1 (molar ratio 1.5:1) in PBS (pH 7.4) at a constant temperature for 24 h to allow the maleimide to react with the thiol groups. Subsequently, the mixture was dialyzed in pure water using a dialysis bag (molecular weight cut-off 3.0 kDa) for 48 h to remove free DSPE-PEG_2000_-Mal and B6 or Tet1. DSPE-PEG_2000_-B6 and DSPE-PEG_2000_-Tet1 were finally obtained, and their contents were determined using a BCA kit. Finally, DSPE-PEG_2000_-B6 and DSPE-PEG_2000_-Tet1 were incubated with RBCm-NPs (Gen/Don) in a constant temperature water bath for 12 h to obtain B6/Tet1-RBCm-NPs (BTRN), and the unbound peptide chains were removed by ultrafiltration centrifugation.

### 2.6 Statistical analysis

Data are expressed as mean ± standard error of the mean (SEM). Differences between groups were analyzed using Student's *t*-test. A value of *P* < 0.05 was considered statistically significant.

## 3 Results and discussion

### 3.1 Characteristics of BTRN

**Fig. 2:**
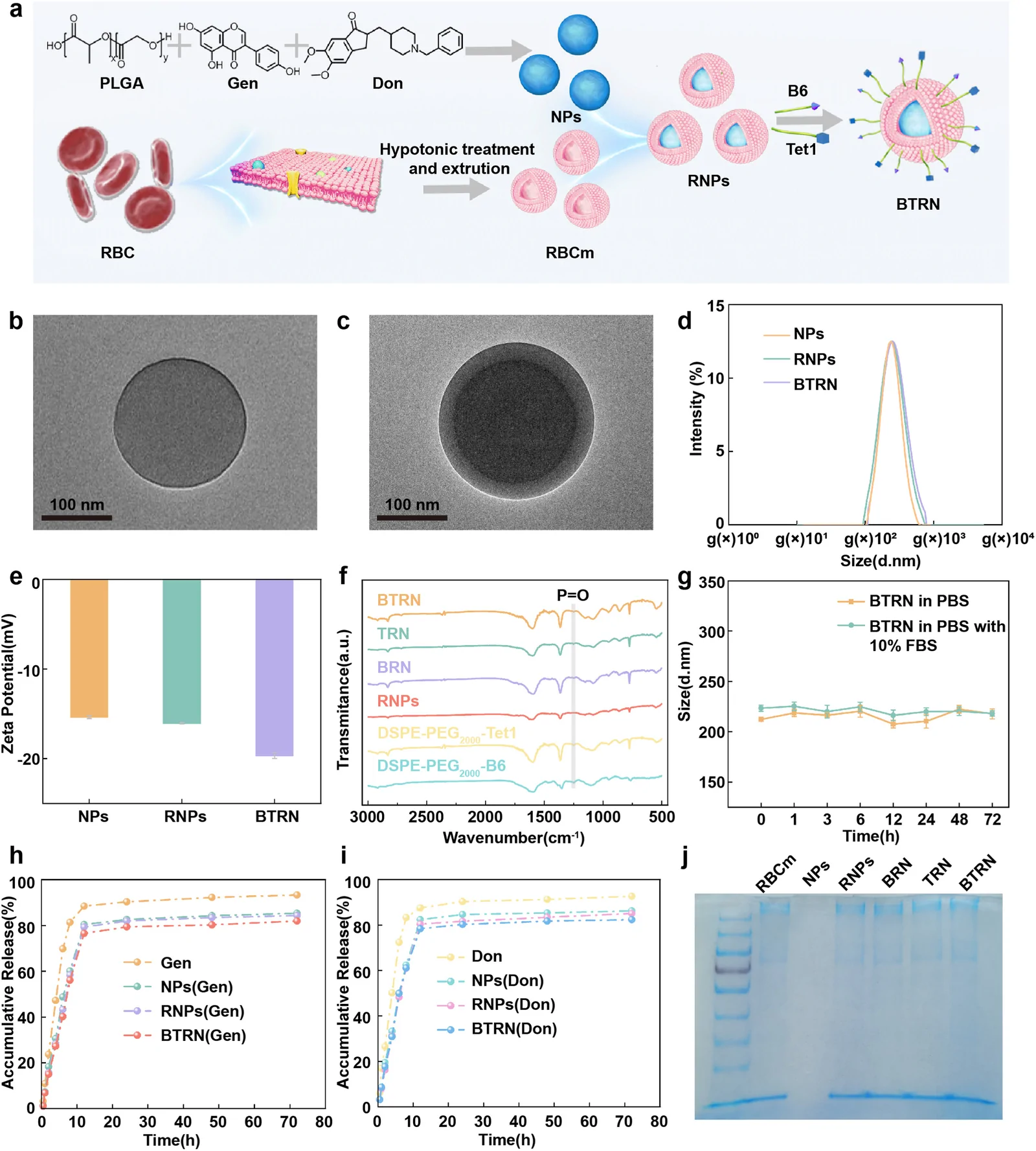
**Characterization of BTRN.** (a) Schematic diagram of the synthetic process of BTRN. (b-c) TEM image of NPs (b) and BTRN (c) (scale bar = 100 nm). (d) Size distribution profiles of NPs, RNPs and BTRN. (e) Zeta-potential values of NPs, RNPs and BTRN. (f) FTIR spectra of DSPE-PEG_2000_-B6, DSPE-PEG_2000_-Tet1, RNPs, BRN, TRN and BTRN. The gray band indicates the characteristic P=O stretching vibration peak at 1240-1260 cm^−1^. (g) Size stability of BTRN in PBS and in PBS with 10% FBS. (h-i) *In vitro* release profiles of Gen (h) and Don (i) from BTRN in PBS (pH7.4) at 37°C. (j) Representative SDS-PAGE protein analysis of RBCm, NPs, RNPs, BRN, TRN, BTRN.

NPs co-loaded with Gen and Don were synthesized through the emulsion solvent evaporation method. A liposome extruder was employed to coat the NPs with RBCm, resulting in RBCm-coated NPs (RNPs). The surface of the RNPs was further modified with the BBB-penetrating peptide B6 and the neuron-targeting peptide Tet1 to obtain BTRN (Fig. 2a). The morphology of the NPs and BTRN was analyzed using TEM (Fig. 2b–c). The BTRN exhibited a spherical shape with a particle size of approximately 220 nm, which is consistent with the results obtained by dynamic light scattering (DLS) (Fig. 2d). All nanoformulations showed negative zeta potentials (Fig. 2e). A higher absolute value of zeta potential generally indicates better system stability, while a moderate negative charge can reduce non-specific electrostatic adsorption between nanomaterials and other negatively charged components in the blood (such as serum proteins and cell membranes), thereby prolonging circulation time. The encapsulation efficiency of the nanocarriers was determined by HPLC. The genistein encapsulation efficiency (EE) exceeded 80% for all three nanoformulations, while the donepezil EE was above 70%. The overall drug loading (DL) capacity was approximately 6% (Table 1).

**Table 1:** Characterization of different nanoparticle formulations.

Formulation	Particle diameters (nm)	Zeta potential (mV)	Gen EE%	Don EE%	DL (Gen + Don) %	PDI
NPs	208.34 ± 1.32	−15.38 ± 0.17	82.34 ± 2.65	74.96 ± 1.32	6.32 ± 0.68	0.235 ± 0.08
RNPs	216.32 ± 1.94	−16.06 ± 0.08	80.76 ± 3.21	72.38 ± 3.65	6.24 ± 0.53	0.227 ± 0.13
BTRN	219.25 ± 2.03	−19.67 ± 0.32	80.12 ± 3.68	71.29 ± 2.94	6.15 ± 0.39	0.241 ± 0.11

To enhance the targeting efficiency, two targeting peptides, DSPE-PEG_2000_-B6 and DSPE-PEG_2000_-Tet1, were used to modify RNPs. DSPE-PEG_2000_-B6 and DSPE-PEG_2000_-Tet1 were synthesized by reacting the thiol groups on the terminal cysteines of B6 and Tet1 with the active group MAL, and their successful synthesis was verified by ^1^H-NMR (Figs. S1–2), with the characteristic peak of thiol at around 1.5 ppm disappearing in the products DSPE-PEG_2000_-B6 and DSPE-PEG_2000_-Tet1. The successful insertion of DSPE-PEG_2000_-B6 and DSPE-PEG_2000_-Tet1 on the surface of RNPs was confirmed by infrared spectroscopy (Fig. 2f), where the P=O stretching vibration of DSPE was observed between 1240 cm^−1^ and 1260 cm^−1^, and the P=O stretching vibration appeared in BRN, TRN, and BTRN, indicating the successful insertion. X-ray photoelectron spectroscopy (XPS) revealed a distinct N 1s peak at approximately 400 eV in the peptide-modified nanoparticles, which was completely absent in the spectrum of the unmodified nanoparticles (Fig. S3). Quantitative elemental analysis demonstrated a marked increase in surface nitrogen atomic concentration, directly attributable to the amide bonds and amino groups of the grafted peptide (Table S1). This emergence and significant enhancement of the nitrogen signal unambiguously confirm the successful conjugation of the peptide onto the nanoparticle surface. Unreacted peptide chains were removed by ultrafiltration centrifugation, and the unreacted peptide chains were quantitatively detected using a BCA kit. The conjugation efficiency of peptides in BTRN was 80.39%.

Given that stability is a prerequisite for *in vivo* application, this study evaluated the aggregation of the biomimetic nanosystems in PBS and PBS supplemented with 10% fetal bovine serum at 37°C for 72 h, the latter condition being employed to simulate the protein-rich environment of blood circulation (Fig. 2g). The results showed that BTRN exhibited good stability and no aggregation occurred.

In addition, *in vitro* release studies of Gen and Don were conducted to investigate the drug release characteristics of the biomimetic nanocarrier system. As shown in Fig. 2h–i, more than 70% of free Gen and Don were released within 6 h. In contrast, NPs, RNPs, and BTRN demonstrated sustained release behavior, with the initial release peak detected at 12 h. This sustained release property helps prevent rapid leakage of the drug during *in vivo* delivery and enhances drug accumulation at the target site. Moreover, the release patterns of NPs, RNPs, and BTRN at each time point showed similar trends, indicating that the encapsulation of RBCm and the modification with targeting peptides had no significant impact on the drug release curve.

To verify the retention and non-destruction of RBCm proteins in the biomimetic nanomedicines, the proteins on the RBCm were analyzed. UV-vis absorption spectra of the erythrocyte membrane-coated nanoparticles displayed a distinct protein-characteristic peak at approximately 280 nm, which was absent in the bare nanoparticles (Fig. S4). The overall spectral profile of the coated nanoparticles closely matched the superimposed spectra of the uncoated core and isolated membrane vesicles, indicating successful transfer of the membrane components. These results confirm that the red blood cell membrane was effectively camouflaged onto the nanoparticle surface. Parallel detection of proteins in different samples was performed by SDS-PAGE. Compared with the RBCm group, cell membrane protein bands were observed in the RNPs, BRN, TRN, and BTRN groups (Fig. 2j), indicating successful encapsulation of RBCm and no damage to the membrane proteins during the modification process. Furthermore, western blotting was employed to specifically detect CD47, a crucial “self-recognition” marker on red blood cells (Fig. S5). CD47 bands were present in all RBCm-containing groups, consistent with the SDS-PAGE findings. This result further confirms that the immune-evasive CD47 protein remained intact and well-preserved on the biomimetic nanomedicines.

### 3.2 Combination index and ratio screening

To determine the optimal molar ratio of BTRN (Gen: Don) against Aβ_25-35_-induced neurotoxicity in HT22 cells, a series of combination ratios was assessed (Fig. 6, Table S2). Cell viability in the model group was 59.36%, while single-agent treatments with BTRN(Gen) and BTRN(Don) restored viability to 77.50% and 71.37%, respectively. Remarkably, combination ratios of 1:1, 2:1, 3:1 and 5:1 (Gen: Don) elevated viability to 88.68%, 85.08%, 82.94% and 79.96%, with corresponding combination index (CI) values of 0.237, 0.396, 0.515 and 0.726, all substantially below 1, indicating robust synergism. In contrast, inverse ratios (1:2, 1:3, 1:5) showed CI values of 1.627, 2.336 and 6.872, accompanied by progressively weaker protection, reflecting antagonistic interactions. Based on the lowest CI value (0.237) and the highest cell viability (88.68%), the 1:1 M ratio of BTRN (Gen: Don) was selected as the optimal combination ratio for further studies.

### 3.3 Safety evaluation and pharmacokinetic profiles

In addition to appropriate physicochemical properties, an ideal nanocarrier should also possess low toxicity and high biocompatibility. Toxicity assays were performed using HT22 (murine hippocampal neuronal cell line for AD-related neuronal injury), hCMEC/D3 (immortalized human brain microvascular endothelial cell line with BBB-like function), and U118 (human glioma cell line for CNS parenchymal biocompatibility assessment) cells. We conducted MTT assays on free Gen + Don, NPs (Gen + Don), RNPs (Gen + Don), BTRN (Gen + Don), and empty BTRN at different concentrations to assess their *in vitro* safety. As shown in Figs. S7–9, after 24-h incubation with different doses of the biomimetic nanosystems, the survival rates of HT22 cells (Fig. S7), hCMEC/D3 cells (Fig. S8), and U118 cells (Fig. S9) were all above 80%, indicating that these biomimetic nanosystems exhibit favorable biocompatibility and negligible cytotoxicity toward CNS-related cellular models, which lays a solid foundation for subsequent *in vivo* AD therapeutic applications.

For *in vivo* safety assessment, mice received daily intravenous injection of different formulations for 14 consecutive days. No overt abnormal behaviors or signs of systemic toxicity were observed throughout the administration period. On day 14, major organs (heart, liver, spleen, lung and kidney) were harvested for histopathological examination via H&E staining. As shown in Fig. S10, compared with the PBS group, no damage indicators were observed in the treated organs, suggesting that intravenous injection of BTRN at the current dosage (5 mg/kg) did not cause systemic toxicity. Additionally, to further validate systemic safety, an additional mouse cohort received daily tail vein injection of BTRN for 14 days, followed by 10-item serum biochemistry and complete blood count (CBC) tests. As summarized in Table S3, no significant differences were observed between the BTRN-treated group and the PBS control group in any of the tested parameters, further supporting the conclusion that BTRN does not induce systemic toxicity at this dose. These results confirms that BTRN have low toxicity and good biocompatibility, which may be attributed to the biodegradable property of the “core” PLGA and the endogenous nature of the RBCm.

Encouraged by the favorable *in vivo* safety profiles, we proceeded to investigate the pharmacokinetic behavior of Gen and Don following a single tail vein injection of either their free forms or the BTRN nanoformulations in mice. As summarized in Table S4, encapsulation of both agents into BTRN nanoparticles dramatically altered their pharmacokinetic parameters. The distribution half-life (t_1/2α_) was prolonged from 0.232 h to 0.640 h for Gen and from 0.283 h to 0.785 h for Don, while the terminal elimination half-life (t_1/2β_) was extended approximately 4- to 5-fold (from 2.361 h to 9.887 h for Gen, and from 2.065 h to 11.532 h for Don). Concurrently, total body clearance (CLz) was reduced by over 90%, dropping from 7.593 to 0.553 L/h/kg for Gen and from 6.832 to 0.537 L/h/kg for Don. Correspondingly, the area under the plasma concentration-time curve (AUC_0-t_) increased by approximately 13- to 14-fold for both drugs, and the mean residence time (MRT_0-t_) was markedly prolonged from 0.502 h to 12.464 h for Gen and from 0.648 h to 14.183 h for Don, confirming significantly extended systemic circulation of the drugs. Collectively, these results confirm that the BTRN nanoformulation confers a long-circulating profile and significantly improved pharmacokinetic properties. For brain-targeted delivery systems, the extended circulation time can provide a sustained concentration gradient and sufficient contact time for nanoparticles to cross the blood-brain barrier, laying a favorable pharmacokinetic foundation for improved brain drug delivery efficiency.

### 3.4 BTRN penetrate the BBB

**Fig. 3:**
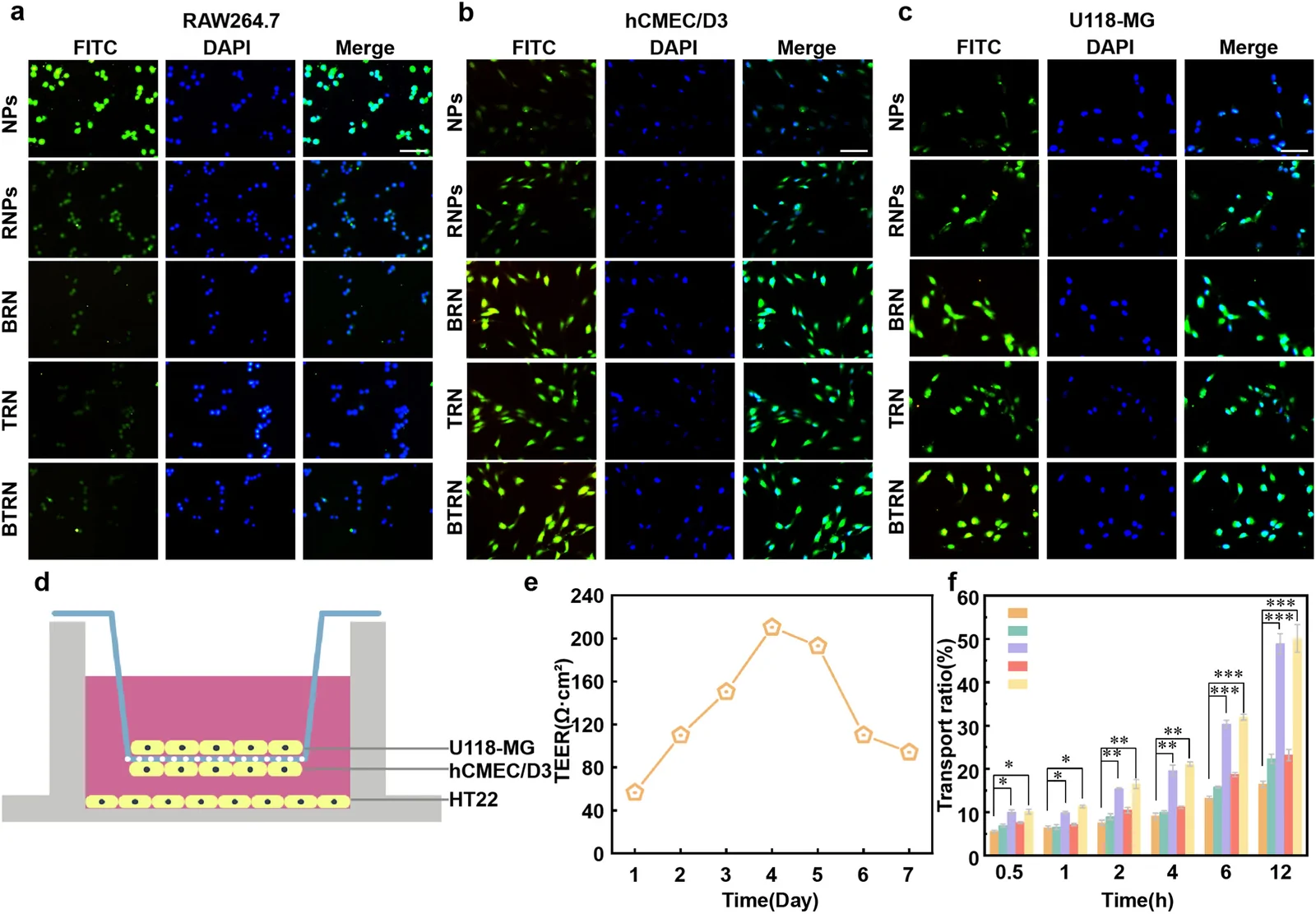
***In vitro* BBB crossing evaluation of the biomimetic NPs.** (a-c) Fluorescence microscopy images of cellular uptake of FITC-labeled NPs in (a) RAW264.7, (b) U-118MG, and (c) hCMEC/D3 cells (scale bar = 200 μm). Blue: DAPI (nuclei) and green: FITC-labeled NPs. (d) Schematic diagram of *in vitro* BBB co-culture model. (e) Time-dependent variation of TEER values in the BBB co-culture model over a 7-day period. (f) Transport efficiency of NPs, RNPs, BRN, TRN and BTRN across *in vitro* BBB model at different time points (n = 3). *p < 0.05, **p < 0.01, ***p < 0.001, compared with NPs group. (For interpretation of the references to color in this figure legend, the reader is referred to the Web version of this article.)

To verify the immune evasion capacity of BTRN, cellular uptake assays were performed using RAW264.7 cells—a widely used murine macrophage cell line that recapitulates the phagocytic activity of the mononuclear phagocyte system (Fig. 3a, Fig. S11). The results showed that after being wrapped with RBCm, the fluorescence intensity of the cellular uptake of each group of nanomaterials by RAW264.7 cells was significantly weakened, confirming the immune evasion ability of the RBCm-encapsulated nanodrug delivery system.

Meanwhile, the brain-targeting ability of BTRN was investigated by performing cellular uptake experiments using hCMEC/D3 and U118 cells (Fig. 3b–c, Figs. S12-13). The results indicated that the modification with B6 targeting peptide significantly increased the cellular uptake of the nanocarriers by hCMEC/D3 and U118 cells. Additionally, an *in vitro* BBB model was established to evaluate the BBB penetration rate of BTRN (Fig. 3d). By seeding U118, hCMEC/D3, and HT22 cells in Transwell chambers and measuring their impedance values, a value above 180 Ω cm^2^ was considered as successful model establishment.

As shown in Fig. 3e, the impedance value peaked at 210.37 ± 1.14 Ω cm^2^ on day 4 of model establishment, confirming successful formation of a functional *in vitro* BBB model. Subsequently, the BBB permeability was determined using this model. As shown in Fig. 3f, following 12 h treatment, the BBB penetration rate of the B6-modified NPs group was significantly higher than those of the NPs group and the RNPs group. At 12 h, the penetration rate of the NPs group was 16.43 ± 0.75%, while that of the BTRN group reached 50.09 ± 3.24%, indicating that surface modification with the B6 targeting peptide significantly enhanced the BBB crossing capacity of the nanodrug delivery system.

**Fig. 4:**
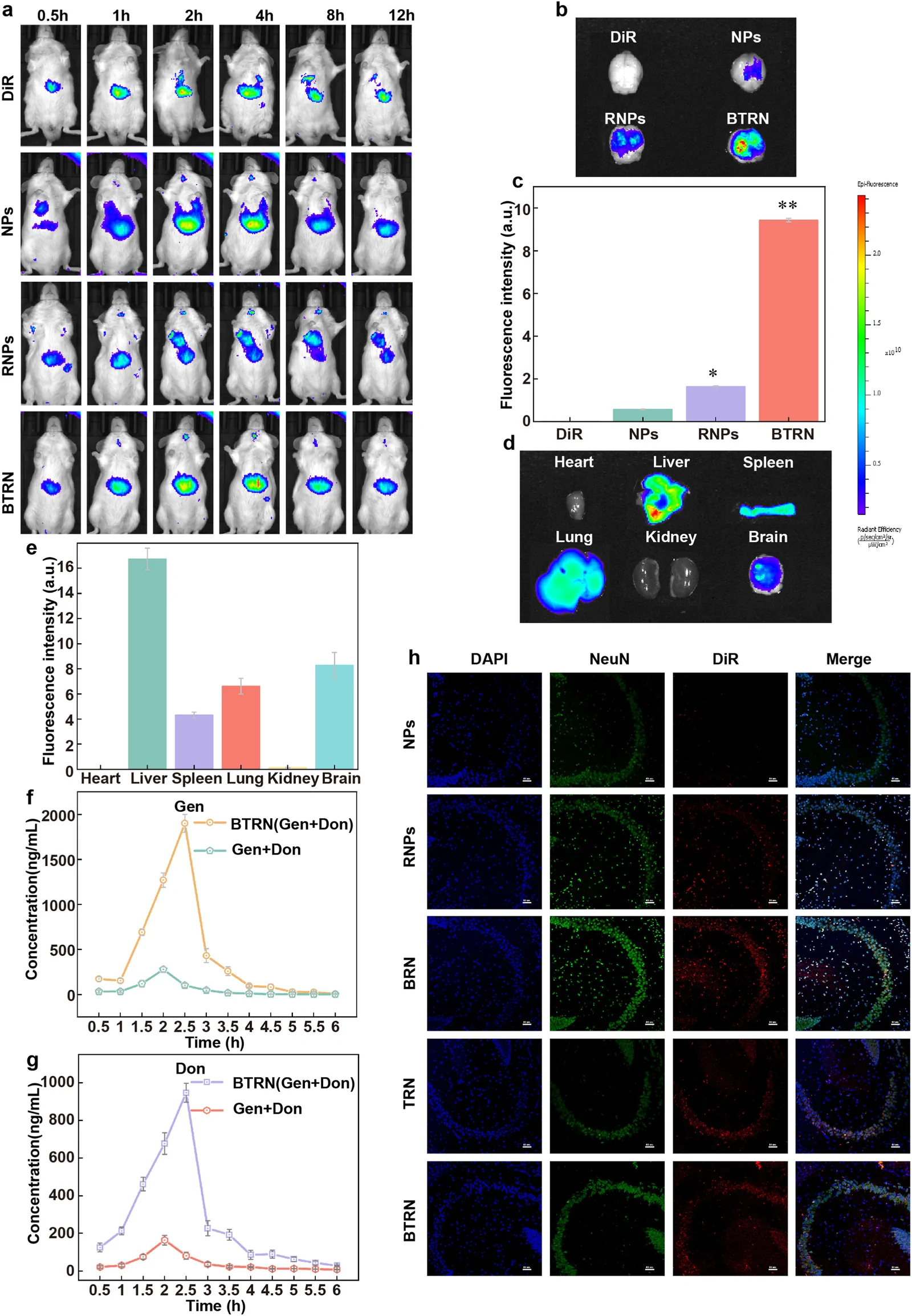
***In vivo* BBB transcytosis and neuronal targeting efficacy of biomimetic NPs.** (a) *In vivo* fluorescence imaging of free DiR, NPs, RNPs, BTRN in mice at different time points post-intravenous injection. (b-e) *Ex vivo* fluorescence images of mouse brain (b) and major organs (d) harvested at 4 h post-injection. Quantitative analysis of DiR fluorescence intensity in brain (c) and major organs (e) *p < 0.05 and **p < 0.01 compared with NPs (n = 3). (f-g) Microdialysis liquid chromatography-mass spectrometry analysis of Gen (f) and Don (g) in brain tissue within 6 h after tail vein injection of BTRN (Gen + Don) (n = 3). (h) Immunofluorescence images of NPs, RNPs, BRN, TRN, and BTRN (red) in the CA3 region of the mice hippocampus (scale bar = 50 μm), blue: DAPI (nuclei) and green: NeuN (neuronal). (For interpretation of the references to color in this figure legend, the reader is referred to the Web version of this article.)

Furthermore, to investigate the *in vivo* brain targeting efficacy of the nanocarriers, DiR fluorescent probes were used to coat the nanocarriers instead of drugs. The nanodrugs of each group were injected into mice via the tail vein, and fluorescence imaging was performed at 1, 2, 4, 6, 8, and 12 h post-injection (Fig. 4a). *In vivo* fluorescence imaging results revealed that the fluorescence intensity in the mouse brain peaked at 4 h post-injection. Compared with the DiR group, NPs group, and RNPs group, the fluorescence intensity of the BTRN group was significantly increased. At 4 h after injection, brains and other major organs were harvested for ex vivo imaging. The DiR fluorescence signal in the brain of the BTRN group was approximately 4.5-fold higher than that of the RNPs group (Fig. 4b–c). Additionally, ex vivo fluorescence imaging of the heart, liver, spleen, lung, kidney, and brain was performed. As shown in Fig. 4d–e, the nanocarriers were predominantly accumulated in the liver, which suggests that the liver may act as the principal organ responsible for the metabolism and clearance of these nanocarriers.

To accurately determine the drug content in the hippocampus of the brain, we used microdialysis *in vivo* sampling technology, with the hippocampus of the mice brain as the sampling site. The probe with a semi-permeable membrane tip was implanted into the mice hippocampus (−2, −2, −2) using a stereotactic apparatus. After the surgery, the mice were allowed to recover for 3-5 days. Before sampling, the *in vivo* recovery rates of the detection probes for Gen and Don were 29.58 ± 0.84% and 28.76 ± 1.23%, respectively. The drugs were administered via tail vein injection (10 mg/kg), and samples were collected every 0.5 h after administration. Cerebrospinal fluid samples were continuously collected for 6 h through the probe, and Gen and Don in the free Gen and Don group and the BTRN (Gen + Don) group were detected by LC-MS (Fig. 4f–g). The results indicated that BTRN significantly increased the accumulation concentration of drugs in the hippocampus and prolonged the drug duration.

### 3.5 BTRN selectively target neuron

AD lesions predominantly occur in hippocampus and entorhinal cortical neurons. Therefore, the specific targeting of neurons within AD lesions by BTRN is crucial for achieving optimal therapeutic efficacy. To verify this targeting capacity, FITC fluorescent probes were loaded onto nanocarriers instead of drugs and added to the apical chamber of the *in vitro* BBB co-culture model, followed by assessment of cellular uptake by HT22 cells seeded in the basolateral chamber of the transwell plate. As shown in Figs. S14 and S15, the fluorescence intensity of the TRN and BTRN groups was significantly higher than that of the other groups, which proved the neuronal targeting capability of the Tet1 peptide.

To further confirm the neuronal targeting capability of BTRN, we replaced the drug with the DiR fluorescent probe and administered it to mice via tail vein injection. Brain tissues were subsequently harvested for frozen sectioning, and neurons were immunolabeled with NeuN to observe the distribution of NPs in the brain. Notably, the hippocampal CA3 subregion was selected as a key observation site, as it is particularly vulnerable to Aβ aggregation and neuronal loss in AD pathogenesis, representing a primary locus of early AD-associated neuropathological damage. As shown in Fig. 4h and Fig. S16, compared with the NPs or RNPs groups, a stronger fluorescence signal was observed in the CA3 region of the hippocampus in the BTRN group, which was attributed to the selective binding of Tet1 to the highly expressed GT1b ganglioside and sphingomyelin in neurons. Collectively, these findings demonstrate that BTRN effectively crosses the BBB and preferentially accumulates within neurons in AD-prone brain regions.

### 3.6 BTRN improves the motor ability and cognitive impairment of APP/PS1 mice

**Fig. 5:**
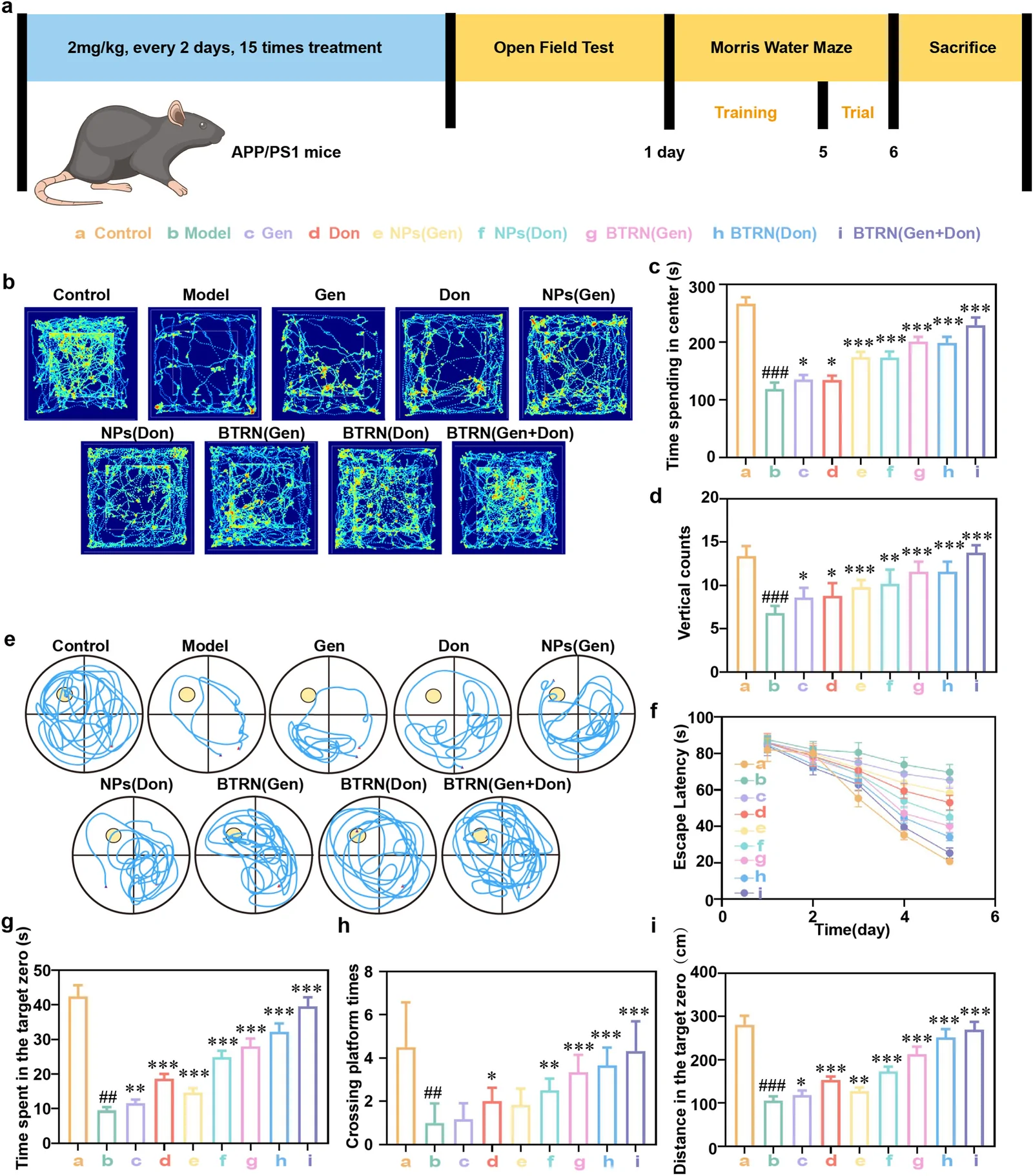
**Behavioral tests of APP/PS1 mice treated with biomimetic NPs.** (a) Timeline of the animal experiment. (b) Representative locomotor trajectories of mice from nine groups in the OFT. (c) Duration of central zone occupancy and (d) vertical rearing counts of APP/PS1 mice in the OFT (n = 6). (e) Representative swimming trajectories of mice in the MWM. (f) Escape latency, (g) duration of target quadrant occupancy, (h) number of platform crossing, and (i) travel distance in the target quadrant of APP/PS1 mice in the MWM. The data are presented as mean ± SEM (n = 6). ##*p* < 0.01, ###*p* < 0.001, compared with the control group. *p < 0.05, **p < 0.01, ***p < 0.001, compared with model group.

To evaluate the *in vivo* therapeutic effect of NPs, 6-month-old APP/PS1 transgenic mice (a well-established murine model of AD) received tail vein injections every 2 days for a 1-month treatment period (Fig. 5a). The administration dose was set at 2 mg/kg, with all doses calculated based on the content of the raw drug. Specifically, the BTRN (Gen + Don) group was 2 mg of Gen and Don, and the effective drugs in the other groups were all 2 mg. The Open Field Test (OFT) is a well-validated behavioral assay widely used to assess non-cognitive behavioral deficits in APP/PS1 mice, particularly alterations in spontaneous activity and exploratory behavior. These behavioral changes are often early manifestations of AD and may occur even before obvious memory loss, which is crucial for a comprehensive understanding of the pathological process of AD and the evaluation of therapeutic drugs. Therefore, the OFT was performed to compare spontaneous activity and exploratory behavior among mice following the 1-month treatment regimen.

**Fig. 6:**
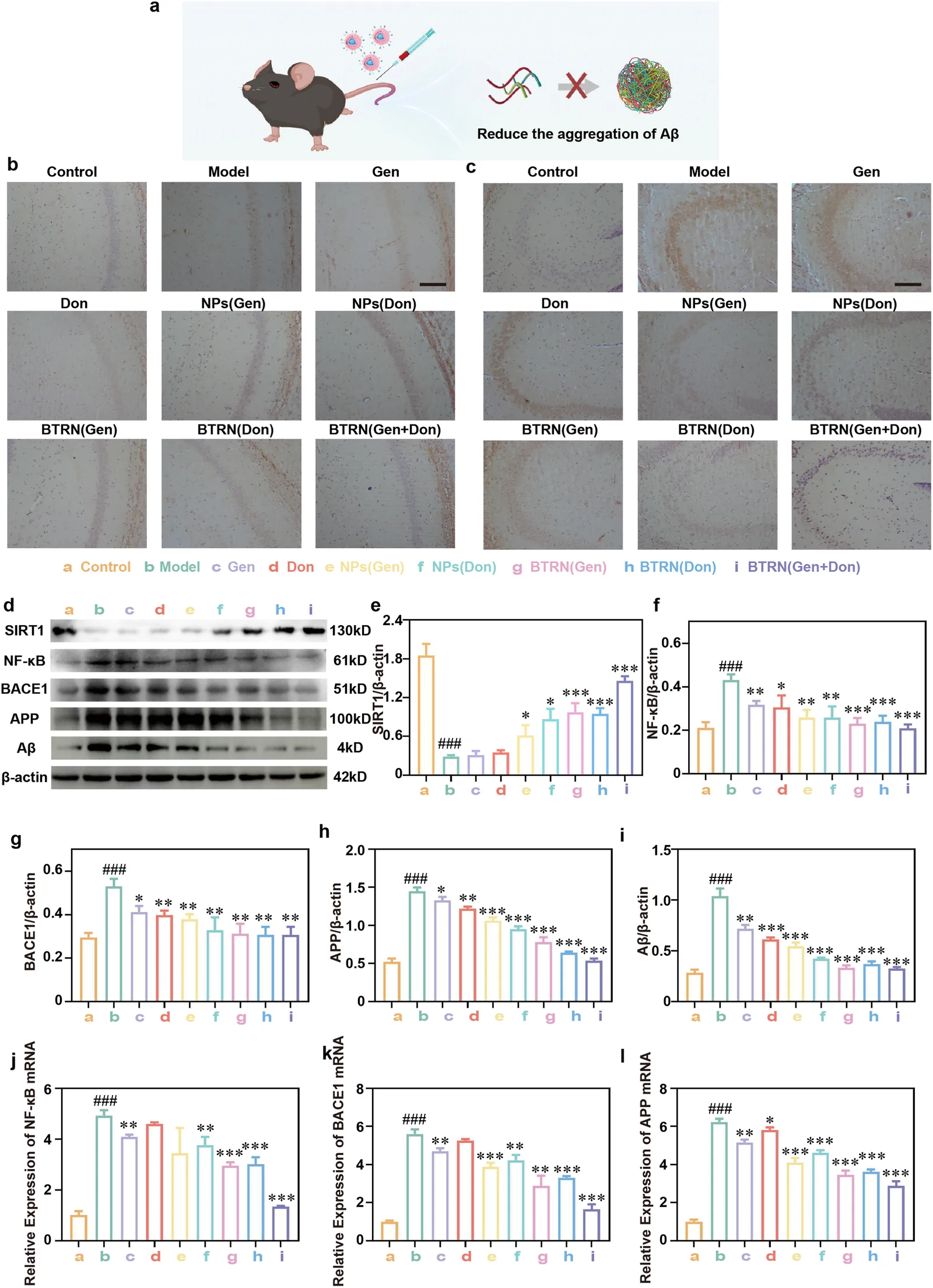
**BTRN modulates Aβ production and the NF-κB/BACE1/APP signaling pathway in APP/PS1 mice.** (a) Schematic illustration of the proposed mechanism by which BTRN reduces the generation of Aβ. (b-c) Representative IHC staining images showing Aβ deposition in the hippocampus CA1 (b) and CA3 (c) subregions of APP/PS1 mice (scale bar = 50 μm). (d-h) Representative WB images (d) and corresponding quantification results for SIRT1 (e), NF-κB (f), BACE1 (g), APP (h) and Aβ (i). (j-l) The mRNA expression levels of NF-κB, BACE1 and APP were quantified using real-time qPCR. Data were presented as mean ± SEM (n = 3). ###*p* < 0.001, compared with the control group. *p < 0.05, **p < 0.01, ***p < 0.001, compared with model group.

Compared with the control group, AD model mice exhibited significantly shorter duration of stay and shorter travel distance in the central zone of the open field, with a distinct tendency to remain near the peripheral walls of the arena. Their avoidance of the open area reflected an increased fear of the potentially dangerous environment. In contrast, the BTRN (Gen + Don) group significantly improved the spontaneous activity of APP/PS1 mice (Fig. 5b–c), increasing the time spent and the distance moved in the central area. At the same time, the frequency of rearing behavior (hindlimb standing) in APP/PS1 mice was quantified (Fig. 5d). Rearing behavior is a well-recognized indicator of active vertical exploratory activity in rodents; the decreased rearing frequency observed in AD model mice reflects diminished curiosity and exploratory motivation. Notably, BTRN (Gen + Don) treatment led to a significant increase in the rearing frequency of APP/PS1 mice. Collectively, these OFT results demonstrate that BTRN (Gen + Don) can effectively reverse the AD-associated impairments in spontaneous locomotor activity and exploratory behavior.

The Morris water maze (MWM), a behavioral paradigm in which mice are required to swim and learn to locate a submerged escape platform, is widely regarded as the gold-standard assay for evaluating spatial learning and memory in rodents. Therefore, the MWM test was performed to assess the spatial learning and memory performance of APP/PS1 mice. As shown in Fig. 5e, the typical swimming paths of each group of mice were recorded. Compared with other groups, AD mice treated with BTRN (Gen + Don) showed significantly shorter escape latency to locate the hidden platform (Fig. 5f), spent a markedly longer duration in the target quadrant (Fig. 5g), crossed the platform location more frequently (Fig. 5h), and traveled a greater distance within the target quadrant (Fig. 5i). The results of the MWM experiment indicated that BTRN (Gen + Don) could alleviate the memory impairment of AD mice to a certain extent.

Notably, while both BTRN (Gen) and BTRN (Don) monotherapies exerted favorable therapeutic effects, the co-delivery formulation of BTRN (Gen + Don) yielded significantly superior efficacy-indicating a synergistic therapeutic outcome that exceeded the simple additive effects of the two individual agents. Additionally, during the treatment course, although APP/PS1 mice receiving free Don monotherapy exhibited partial improvements in motor function and cognitive deficits, their overall health status was markedly compromised. This phenomenon was presumably attributed to dose-dependent adverse reactions induced by systemic free Don administration. Importantly, this adverse effect was ameliorated when Don was delivered in the form of nanoparticle-encapsulated formulations. In conclusion, BTRN (Gen + Don) significantly ameliorates motor deficits and cognitive impairment in APP/PS1 mice.

### 3.7 BTRN reduces the generation of Aβ

The abnormal aggregation of Aβ is a central event in the pathogenesis of AD. The resulting neurotoxic Aβ plaques exhibit a strong correlation with cognitive impairment. A key rate-limiting step in aberrant Aβ generation is the cleavage of APP by BACE1. BTRN likely reduces Aβ production via the NF-κB/BACE1/APP pathway (Fig. 6a). Given the established impact of Aβ deposition in the hippocampal CA1 and CA3 regions on spatial learning and memory deficits characteristic of AD, we assessed Aβ accumulation levels in the CA1 region (Fig. 6b) and CA3 region (Fig. 6c) of the hippocampus of APP/PS1 mice using immunohistochemical (IHC) staining. The results showed that the number of Aβ-positive plaques in the hippocampal CA1 and CA3 subregions was significantly increased in the AD model group than in the control group. This increase was markedly attenuated by treatment with BTRN (Gen + Don). These findings were supported by quantitative data (Figs. S17–18), which closely aligned with the representative images.

To further verify the therapeutic effect of BTRN (Gen + Don) against Aβ induced neurotoxicity, we first established an *in vitro* AD cell model using Aβ_25-35_—a neurotoxic fragment of full-length Aβ that is widely recognized for its ability to induce neuronal oxidative stress, apoptosis, and viability loss, mimicking the pathological damage observed in AD brains. The MTT assay was then employed to evaluate the protective effects of different drug treatment groups on HT22 hippocampal neuronal cell viability impaired by Aβ_25-35_ exposure. As shown in Fig. S19, BTRN (Gen + Don) significantly alleviated the cell viability damage caused by Aβ_25-35_ induction.

Based on previous experimental findings, we hypothesized that the suppression of Aβ generation by BTRN may be attributed to Gen-mediated SIRT1 activation, which sequentially inhibits NF-κB activation, downregulates the expression of BACE1, the rate-limiting enzyme for Aβ production, and reduces the cleavage of APP, thereby reducing Aβ production. To verify this putative mechanism, we performed WB assays. As shown in Fig. 6d, BTRN treatment significantly upregulated the protein expression of SIRT1 while markedly reducing the levels of NF-κB, BACE1, APP, and Aβ. Densitometric quantification of the corresponding protein bands further confirmed these changes (Fig. 6e–i).

Additionally, the mRNA expression levels of NF-κB, BACE1, and APP were determined by quantitative real-time-PCR (qRT-PCR) (Fig. 6j–l). Compared with the control group, the mRNA levels of these three genes were significantly elevated in the model group, while BTRN treatment markedly downregulated the mRNA expression of NF-κB, BACE1, and APP in APP/PS1 mice. Consistent with the aforementioned WB results, these findings verified that BTRN suppresses Aβ production via modulating the NF-κB/BACE1/APP signaling pathway.

To further confirm the indispensable role of NF-κB in this regulatory mechanism, we employed NF-κB activator 1 (compound 32) to specifically stimulate NF-κB in Aβ_25-35_-induced HT22 cells. As shown in Figs. S20–22, activation of NF-κB by compound 32 almost completely abolished the BTRN-induced reduction in BACE1 and Aβ protein levels. In contrast to the pronounced decrease observed with BTRN treatment alone, co-treatment with the NF-κB agonist resulted in BACE1 and Aβ levels that no longer followed a declining trend, indicating that the suppressive effects of BTRN on these proteins are critically NF-κB-dependent.

### 3.8 BTRN improves the decline of cholinergic system function

**Fig. 7:**
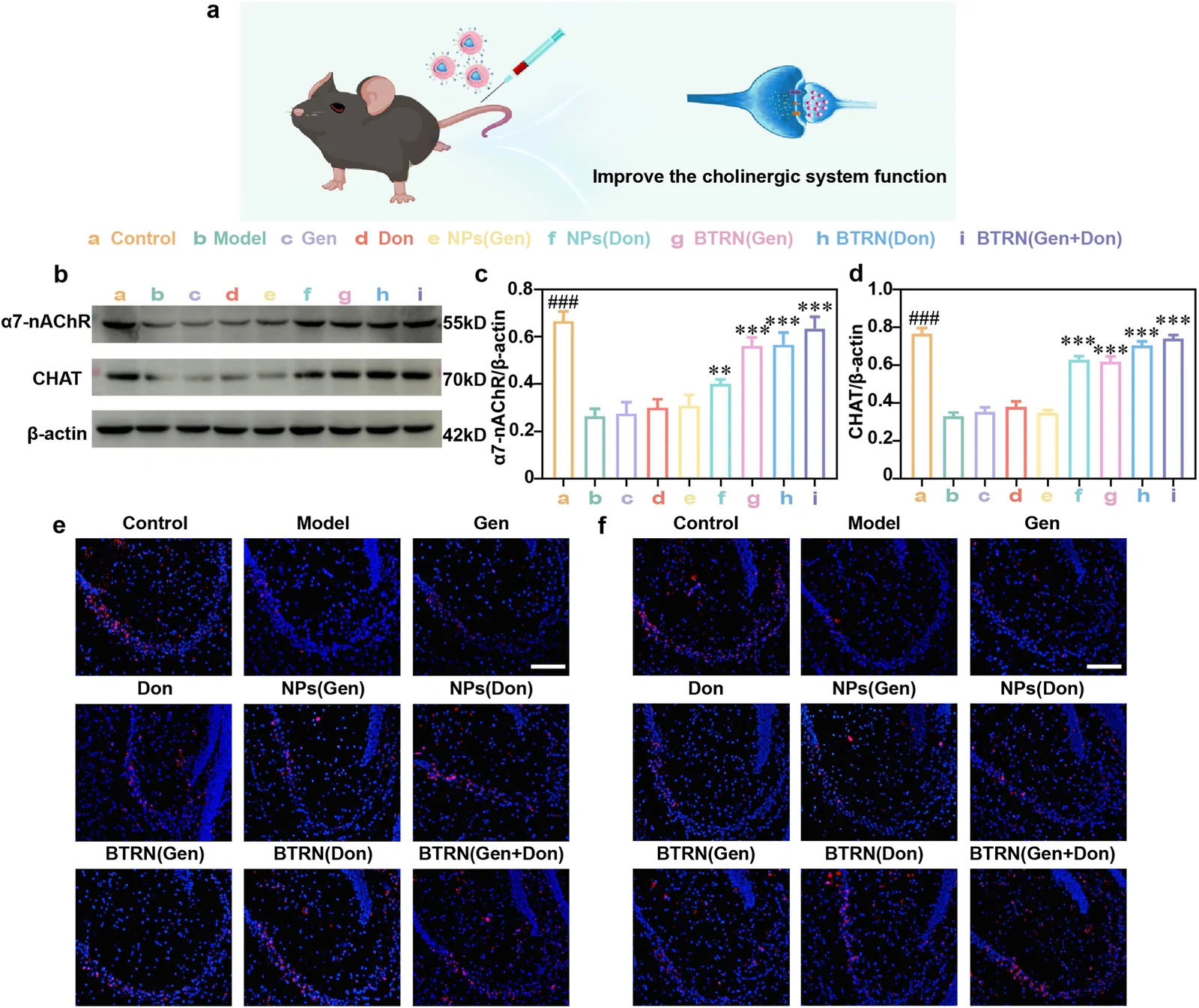
**BTRN ameliorates cholinergic system dysfunction in APP/PS1 mice.** (a) BTRN improves the decline of cholinergic system function. (b-d) Representative WB images (b) and corresponding quantification of α7-nAChR (c) and CHAT (d) expression. (e-f) Representative immunofluorescent images of α7-nAChR (e) and CHAT (f) in hippocampus CA3 subregions of APP/PS1 mice (scale bar = 50 μm). Data were presented as mean ± SEM (n = 3). ###*p* < 0.001, compared with the control group. *p < 0.05, **p < 0.01, ***p < 0.001, compared with model group.

Donepezil is a well-established drug for AD treatment, whose main mechanism of action is to block acetylcholine hydrolysis by cholinesterase, alleviate neuronal excitotoxicity, and thereby improve the decline of cholinergic system function (Fig. 7a). α7nAChR is one of the key receptors for acetylcholine and is highly enriched in the learning and memory centers of the brain (such as the hippocampus). CHAT is an essential enzyme for the production of acetylcholine and its distribution is almost parallel to that of acetylcholine. Based on this, we determined the protein content of α7nAChR and CHAT via WB assays (Fig. 7b–d). The results demonstrated that BTRN treatment significantly upregulated the protein expression levels of α7nAChR and CHAT, indirectly verifying the increase in acetylcholine levels. Meanwhile, immunofluorescence staining was performed to visualize the localization of α7nAChR and CHAT in the hippocampal CA3 region (Fig. 7e–f, Figs. S23-24). These results were consistent with those of the WB assays, proving that BTRN enhances cholinergic system function by blocking the hydrolysis of acetylcholine by cholinesterase.

To further directly quantify hippocampal acetylcholine abundance and validate the cholinergic-protective effect of BTRN at the functional level, we detected extracellular ACh concentrations in freely moving mice using microdialysis combined with LC-MS. All drug treatments were administered via tail vein injection at a dose of 2 mg/kg every other day for 30 consecutive days. The control and model groups received an equivalent volume of saline via the same route and on the same schedule. The basal ACh concentration in the dialysate of wild-type control mice was 3.47 ± 0.33 nM. In contrast, 6-month-old male APP/PS1 model mice exhibited a significant 47.6% reduction in ACh, dropping to 1.82 ± 0.27 nM. Don alone (2 mg/kg) partially rescued this deficit, elevating ACh to 2.77 ± 0.32 nM. The free combination of Gen + Don (total dose 2 mg/kg) produced a more pronounced effect, reaching 2.94 ± 0.21 nM. Notably, BTRN (Gen + Don) restored hippocampal ACh to 3.39 ± 0.45 nM, a level statistically indistinguishable from that of the control group. The BTRN group showed a trend toward higher ACh compared with the free Gen + Don combination, suggesting the enhanced therapeutic efficacy of the targeted nanodelivery system. These results demonstrate that BTRN effectively reinstates cholinergic neurotransmission in the hippocampus of APP/PS1 mice.

### 3.9 BTRN reduces neuronal apoptosis

**Fig. 8:**
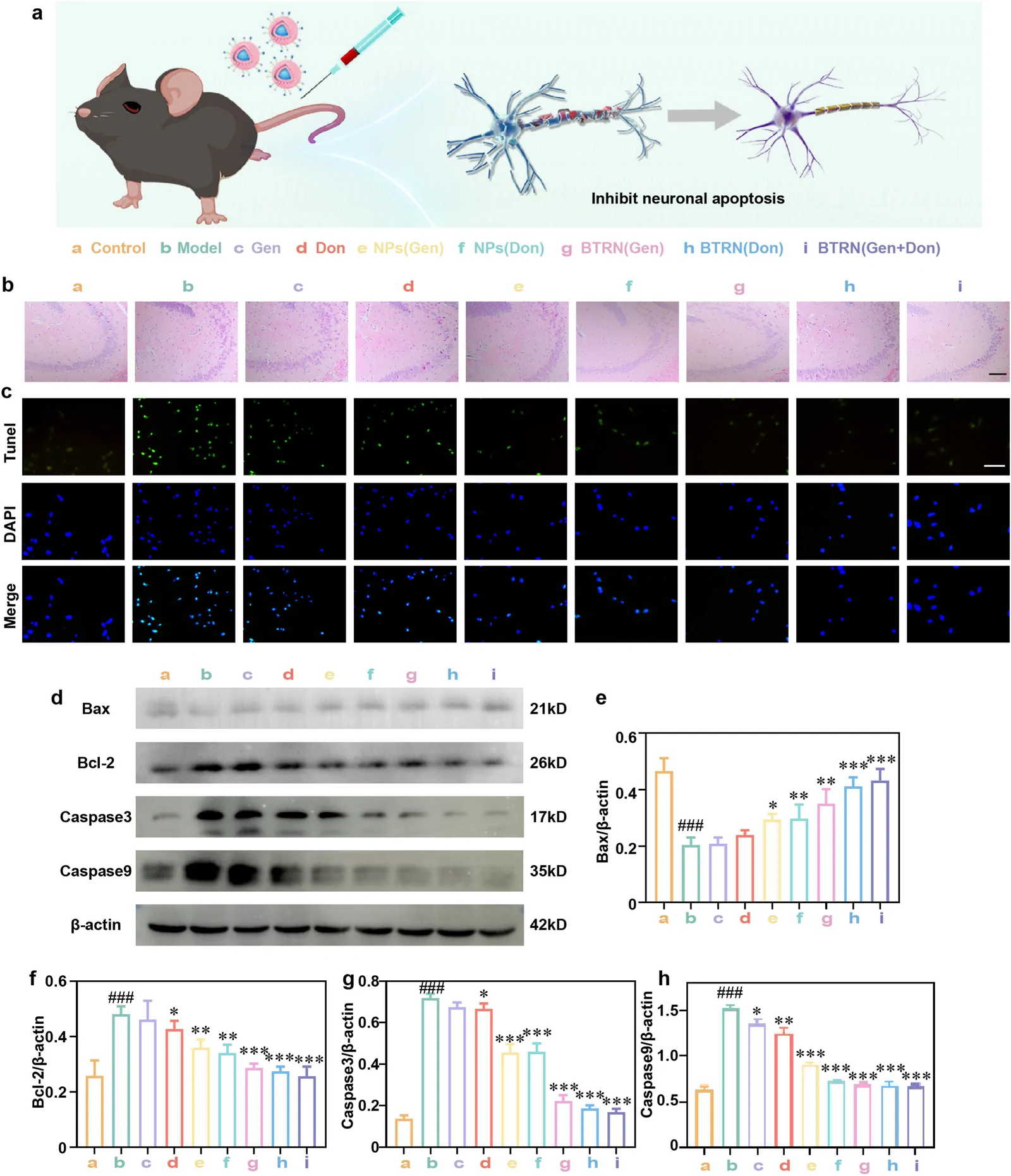
**BTRN attenuates neuronal apoptosis in APP/PS1 mice.** (a) BTRN reduces neuronal apoptosis. (b) HE staining images of the hippocampal CA3 subregion in APP/PS1 (scale bar = 50 μm). (c) Representative TUNEL staining images of apoptotic neurons (scale bar = 200 μm). (d-h) Representative WB images (d) and corresponding quantification of Bax (e), Bcl-2(f), caspase 3 (g) and caspase 9 (h) expression. Data were presented as mean ± SEM (n = 3). ###*p* < 0.001 compared with the control group. *p < 0.05, **p < 0.01, ***p < 0.001, compared with model group.

Neuronal apoptosis represents the final and most critical step in the pathological cascade of AD, leading to irreversible brain damage and neurological dysfunction. Its significance is closely correlated with other core pathological hallmarks of AD rather than being an isolated event, and these pathologies collectively drive progressive cognitive decline. Therefore, the capacity of BTRN to inhibit neuronal apoptosis is crucial for AD treatment (Fig. 8a). We examined the structural damage of neurons in the CA1 region (Fig. S25), CA3 region (Fig. 8b), and dentate gyrus (Fig. S26) of the hippocampus of APP/PS1 mice via HE staining. The HE staining results showed that the cells in the hippocampus of the control group mice were arranged neatly, with a large number of neurons, uniform staining, and clear nucleoli; while in the model group mice, the hippocampal cells were disordered, some neurons were lost, the cells were shrunken, and the nucleoli were not obvious. Treatment with Gen- and/or Don-loaded formulations alleviated neurodegenerative changes in the hippocampi of APP/PS1 mice. The trend of improvement in the hippocampal structure in all treatment groups was consistent with the trend of reversing cognitive deficits shown in Fig. 5. Particularly, BTRN (Gen + Don) treatment significantly mitigated neuronal damage, as evidenced by the intact morphology, orderly arrangement, and uniform color of the neurons.

Next, we employed TUNEL staining, a classic cytochemical technique for labeling apoptotic cells, to quantify the number of apoptotic neurons. In the TUNEL assay, we used 20 μmol/L Aβ_25-35_ to model HT22 cells, and then incubated each group of nanodrugs with the cells for 24 h. As shown in Fig. 8c, compared with the control group, the fluorescence intensity of TUNEL-positive neurons was markedly elevated in the Aβ-induced group, while the treatment with BTRN (Gen + Don) significantly decreased the proportion of TUNEL-positive neurons.

To reveal the detailed molecular mechanism underlying this anti-apoptotic effect, we next investigated the mitochondrial-dependent intrinsic apoptotic pathway by detecting the subcellular distribution of cytochrome *c* (Cyt *c*). Immunofluorescence staining for Cyt *c* revealed that in Aβ_25-35_-induced HT22 cells, Cyt *c* exhibited a diffuse cytosolic distribution, indicative of its release from mitochondria, whereas BTRN treatment largely restored punctate Cyt *c* signals distributed along spiral mitochondrial filaments and confined within intact mitochondria (Fig. S27). Consistently, Western blot analysis demonstrated that BTRN significantly upregulated the anti-apoptotic protein Bcl-2 and downregulated the pro-apoptotic protein Bax, leading to an increased Bcl-2/Bax ratio (Fig. 8d–f). These results suggest that BTRN stabilizes mitochondrial integrity by preserving the balance between pro- and anti-apoptotic Bcl-2 family members, thereby preventing Cyt *c* release. As a downstream consequence, we assessed the expression of caspase 9 and caspase 3, two key mediators of the intrinsic apoptotic cascade, via Western blotting (Fig. 8g–h). BTRN treatment markedly reduced the levels of cleaved caspase 9 and cleaved caspase 3, indicating that BTRN suppresses the activation of these initiator and executioner caspases, blocks the apoptotic signaling cascade, and ultimately mitigates neuronal apoptosis.

## 4 Discussion

The complex pathophysiology of AD, which involves interconnected pathological cascades including Aβ aggregation, cholinergic dysfunction, and neuronal apoptosis, increasingly demands therapeutic strategies that extend beyond monotargeted interventions. The BTRN biomimetic nanoplatform developed in this study addresses this clinical need through the rational combination of two mechanistically complementary agents: the natural product genistein and the established acetylcholinesterase inhibitor donepezil. The synergistic interplay between these two compounds represents a cornerstone of the therapeutic design. Genistein, a phytoestrogen with pleiotropic bioactivities, has demonstrated efficacy in reducing Aβ production by modulating amyloid precursor protein processing and promoting non-amyloidogenic pathways. Concurrently, donepezil provides rapid and sustained restoration of cholinergic neurotransmission, directly addressing the cognitive decline associated with cholinergic neuron loss. Beyond their primary mechanisms, both agents exhibit anti-apoptotic properties that collectively enhance neuronal survival, suggesting a multifaceted cooperative effect that is particularly advantageous for targeting the heterogeneous pathology of AD.

The synergistic efficacy of genistein and donepezil is further amplified by their differential yet overlapping molecular targets. Genistein's ability to inhibit oxidative stress and neuroinflammation complements donepezil's cholinergic enhancement, creating a therapeutic environment that simultaneously addresses multiple drivers of disease progression. This rational pairing exemplifies the principle that combination therapies, when designed with careful consideration of agent selection and dosing regimens, can achieve enhanced therapeutic outcomes while potentially mitigating the dose-dependent side effects associated with high-dose monotherapy. Importantly, the co-encapsulation of these agents within a single nanocarrier ensures synchronized pharmacokinetics and spatially coordinated delivery to diseased neurons, maximizing the probability of synergistic interaction at the cellular level. This approach circumvents the pharmacokinetic mismatches often encountered with simple drug cocktails, where differential absorption, distribution, and clearance can disrupt the desired synergistic ratio.

The delivery of this synergistic payload to its intended site of action is enabled by the biomimetic and targeting features of the BTRN platform. The PLGA core provides a clinically translatable, biocompatible matrix for the controlled co-release of genistein and donepezil, leveraging the well-established safety profile of this polymer in FDA-approved formulations. Encapsulation within endogenous red blood cell membranes confers immune-evasive properties, prolonging circulation time and enhancing the probability of CNS accumulation. The subsequent functionalization with the B6 peptide facilitates efficient BBB penetration, while the Tet1 peptide mediates specific binding to neuronal cells. This dual-targeting strategy ensures that the synergistic drug combination is preferentially delivered to the neurons most affected by AD pathology, thereby concentrating therapeutic activity at the disease site while minimizing systemic exposure and potential off-target effects.

In a word, the BTRN system integrates the synergistic potential of natural product-derived and marketed pharmaceuticals within a sophisticated, biomimetic delivery vehicle. By simultaneously targeting Aβ pathology, cholinergic dysfunction, and apoptotic pathways through the combined actions of genistein and donepezil, this platform offers a multifaceted approach that aligns with the complex etiology of AD. The rational selection of complementary therapeutic agents, coupled with advanced delivery technologies that ensure precise CNS and neuronal targeting, positions BTRN as a promising strategy for enhancing therapeutic outcomes in AD. This work provides a foundation for further preclinical development and underscores the value of integrating synergistic drug combinations with biomimetic nanocarrier design for the treatment of complex neurodegenerative disorders.

## 5 Conclusion

In summary, we have developed a multifunctional biomimetic NPs-based combinatorial therapeutic platform, which delivers Gen and Don into the brain via receptor-mediated endocytosis and enables specific drug accumulation in neurons within AD lesion-prone regions through the Tet1 peptide. Both *in vitro* and *in vivo* experiments have demonstrated that BTRN acts as a nano-scale drug with favorable safety profiles toward cells, the BBB, and multiple visceral tissues and organs. Moreover, BTRN effectively restores the motor dysfunction and cognitive ability of APP/PS1 mice. Mechanistically, Gen carried by the nano-system has been confirmed to reduce Aβ production through BACE1/NF-κB/APP signaling pathway, while Don regulates cholinergic system function by inhibiting acetylcholine hydrolysis. Notably, the co-delivery of Gen and Don exerts a synergistic effect in suppressing neuronal apoptosis. Collectively, these findings indicate that BTRN has a wide range of regulatory effects and offers new possibilities for the treatment of neurodegenerative diseases, including AD.

## CRediT authorship contribution statement

**Yue Na:** Writing – review & editing, Writing – original draft, Visualization, Validation, Methodology, Investigation, Formal analysis, Data curation. **Chi Liu:** Writing – original draft, Investigation, Formal analysis, Data curation. **Xinming Bi:** Software, Methodology, Investigation. **Shumeng Liu:** Writing – review & editing, Methodology, Investigation. **Yue Xing:** Writing – original draft, Visualization. **Bohan Zhang:** Investigation, Formal analysis. **Tongfeng Liu:** Writing – review & editing, Methodology, Investigation. **Ning Zhang:** Writing – review & editing, Supervision, Funding acquisition. **Fang Geng:** Writing – review & editing, Supervision, Resources, Project administration, Funding acquisition.

## Declaration of competing interest

The authors declare that they have no known competing financial interests or personal relationships that could have appeared to influence the work reported in this paper.

## Data Availability

Data will be made available on request.

## References

[1] Liu L., Liu W., Dong X., Sun Y. (2025). A multifunctional theranostic agent based on rhodamine-modified copper-gallic acid nanoparticles targeting Alzheimer's β-amyloid species. Chem. Eng. J..

[2] Yin X., Zhou H., Cao T., Yang X., Meng F., Dai X., Wang Y., Li S., Zhai W., Yang Z., Chen N., Zhou R. (2024). Rational design of dual-functionalized Gd@C_82_ nanoparticles to relieve neuronal cytotoxicity in Alzheimer's disease via inhibition of Aβ aggregation. ACS Nano.

[3] Qian K., Bao X., Li Y., Wang P., Guo Q., Yang P., Xu S., Yu F., Meng R., Cheng Y., Sheng D., Cao J., Xu M., Wu J., Wang T., Wang Y., Xie Q., Lu W., Zhang Q. (2022). Cholinergic neuron targeting nanosystem delivering hybrid peptide for combinatorial mitochondrial therapy in Alzheimer's disease. ACS Nano.

[4] Cai L., Yang C., Jia W., Liu Y., Xie R., Lei T., Yang Z., He X., Tong R., Gao H. (2020). Endo/lysosome‐escapable delivery depot for improving BBB transcytosis and neuron targeted therapy of Alzheimer's disease. Adv. Funct. Mater..

[5] Du B., Zou Q., Wang X., Wang H., Yang X., Wang Q., Wang K. (2025). Multi-targeted engineered hybrid exosomes as Aβ nanoscavengers and inflammatory modulators for multi-pathway intervention in Alzheimer's disease. Biomaterials.

[6] Jung M., Lee S., Park S., Hong J., Kim C., Cho I., Sohn H.S., Kim K., Park I.W., Yoon S., Kwon S., Shin J., Lee D., Kang M., Go S., Moon S., Chung Y., Kim Y., Kim B. (2023). A therapeutic nanovaccine that generates anti‐amyloid antibodies and amyloid‐specific regulatory T cells for Alzheimer's disease. Adv. Mater..

[7] Lai Y., Zhu Y., Xu Z., Hu X., Saeed M., Yu H., Chen X., Liu J., Zhang W. (2020). Engineering versatile nanoparticles for near‐infrared light‐tunable drug release and photothermal degradation of amyloid β. Adv. Funct. Mater..

[8] Quan P., Guo W., Yang L., Cun D., Yang M. (2023). Donepezil accelerates the release of PLGA microparticles via catalyzing the polymer degradation regardless of the end groups and molecular weights. Int. J. Pharm..

[9] Guo Q., Li Y., Xu S., Wang P., Qian K., Yang P., Sheng D., Wang L., Cheng Y., Meng R., Cao J., Luo H., Wei Y., Zhang Q. (2023). Brain-neuron targeted nanoparticles for peptide synergy therapy at dual-target of Alzheimer's disease. J. Contr. Release.

[10] Hwang C., Lee S.Y., Kim H.J., Lee K., Lee J., Kim D.D., Cho H.J. (2021). Polypseudorotaxane and polydopamine linkage-based hyaluronic acid hydrogel network with a single syringe injection for sustained drug delivery. Carbohydr. Polym..

[11] Hampel H., Mesulam M.M., Cuello A.C., Farlow M.R., Giacobini E., Grossberg G.T., Khachaturian A.S., Vergallo A., Cavedo E., Snyder P.J., Khachaturian Z.S. (2018). The cholinergic system in the pathophysiology and treatment of Alzheimer's disease. Brain.

[12] Zhang L., Hou S., Movahedi F., Li Z., Li L., Hu J., Jia Y., Huang Y., Zhu J., Sun X., Zeng L., Liu R., Xu Z.P. (2023). Amyloid-β/Tau burden and neuroinflammation dual-targeted nanomedicines synergistically restore memory and recognition of Alzheimer's disease mice. Nano Today.

[13] Han G., Bai K., Yang X., Sun C., Ji Y., Zhou J., Zhang H., Ding Y. (2022). “Drug‐carrier” synergy therapy for amyloid‐ β clearance and inhibition of Tau phosphorylation via biomimetic lipid nanocomposite assembly. Adv. Sci..

[14] Li W.B., Xu L.L., Wang S.L., Wang Y.Y., Pan Y.C., Shi L.Q., Guo D.S. (2024). Co‐assembled nanoparticles toward multi‐target combinational therapy of Alzheimer's disease by making full use of molecular recognition and self‐assembly. Adv. Mater..

[15] Zott B., Simon M.M., Hong W., Unger F., Chen-Engerer H.J., Frosch M.P., Sakmann B., Walsh D.M., Konnerth A. (2019). A vicious cycle of β amyloid-dependent neuronal hyperactivation. Science.

[16] Han Y., Gao C., Wang H., Sun J., Liang M., Feng Y., Liu Q., Fu S., Cui L., Gao C., Li Y., Yang Y., Sun B. (2021). Macrophage membrane-coated nanocarriers Co-modified by RVG29 and TPP improve brain neuronal mitochondria-targeting and therapeutic efficacy in Alzheimer's disease mice. Bioact. Mater..

[17] Wahby M.M., Mohammed D.S., Newairy A.A., Abdou H.M., Zaky A. (2017). Aluminum-induced molecular neurodegeneration: the protective role of genistein and chickpea extract. Food Chem. Toxicol..

[18] Zhou S., Hu Y., Zhang B., Teng Z., Gan H., Yang Z., Wang Q., Huan M., Mei Q. (2008). Dose-dependent absorption, metabolism, and excretion of genistein in rats. J. Agric. Food Chem..

[19] Nagori K., Pradhan M., Sharma M., Ajazuddin, Badwaik H.R., Nakhate K.T. (2024). Current progress on central cholinergic receptors as therapeutic targets for Alzheimer's disease. Curr. Alzheimer Res..

[20] Zhang H., Zhao Y., Yu M., Zhao Z., Liu P., Cheng H., Ji Y., Jin Y., Sun B., Zhou J., Ding Y. (2019). Reassembly of native components with donepezil to execute dual-missions in Alzheimer's disease therapy. J. Contr. Release.

[21] Kömür M., Kıyan H.T., Öztürk A.A. (2025). Development of donepezil hydrochloride-loaded PLGA-based nanoparticles for Alzheimer's disease treatment. Sci. Rep..

[22] Yang P., Huang Q., Zhang J., Li Y., Gao H., Gu Z. (2024). Natural polyphenolic nanodots for Alzheimer's disease treatment. Adv. Mater..

[23] Feng Q., Wang N., Zhang X., Mei Y., Fu R., Chen J., Yuan X., Yang S., Zhang Z., Zhao H., Wang L. (2023). Nanoparticle cluster depolymerizes and removes amyloid fibrils for Alzheimer's disease treatment. Nano Today.

[24] Park E., Li L.Y., He C., Abbasi A.Z., Ahmed T., Foltz W.D., O'Flaherty R., Zain M., Bonin R.P., Rauth A.M., Fraser P.E., Henderson J.T., Wu X.Y. (2023). Brain‐penetrating and disease site‐targeting manganese dioxide‐polymer‐lipid hybrid nanoparticles remodel microenvironment of Alzheimer's disease by regulating multiple pathological pathways. Adv. Sci..

[25] Du Q., Liu Y., Fan M., Wei S., Ismail M., Zheng M. (2024). PEG length effect of peptide-functional liposome for blood brain barrier (BBB) penetration and brain targeting. J. Contr. Release.

[26] Wang X., Chen S., Xia X., Du Y., Wei Y., Yang W., Zhang Y., Song Y., Lei T., Huang Q., Gao H. (2025). Lysosome‐targeting protein degradation through endocytosis pathway triggered by polyvalent nano‐chimera for AD therapy. Adv. Mater..

[27] Zhang Q., Song Q., Yu R., Wang A., Jiang G., Huang Y., Chen J., Xu J., Wang D., Chen H., Gao X. (2023). Nano‐brake halts mitochondrial dysfunction cascade to alleviate neuropathology and rescue Alzheimer's cognitive deficits. Adv. Sci..

[28] Chen Y., Wei J., Wang L., Cai Q., Yang F., Zhang L., Liu J., Liu Y. (2024). Multi-enzyme Co-expressed ruthenium dioxide nanoparticles activate mitochondrial autophagy and regulate oxidative stress for Alzheimer's disease treatment. Chem. Eng. J..

[29] Liu P., Zhang T., Wu Y., Chen Q., Sun T., Jiang C. (2024). A peptide‐drug conjugate‐based nanoplatform for immunometabolic activation and in situ nerve regeneration in advanced‐stage Alzheimer's disease. Adv. Mater..

[30] Wang Y., Yan C., Qi Y., Zhao R., Wan Y., Lyu Z., Li Y., Wang Z., Li C., Lin Y., Chen Q., Ding S., Shi J. (2025). Harnessing multi-target nanosweepers inhibiting β-amyloid aggregation, scavenging reactive oxygen species and overcoming the blood brain barrier for rescuing Alzheimer's disease. Chem. Eng. J..

[31] Hou T., Nie D., Ding M., Wang C., Mei K., Lu X., Wang X., Tang S., Wu H., Guan P., Zhu W., Hu X. (2025). Multi-target nanocomposites for Alzheimer's treatment via microenvironment modulation and β-amyloid plaque clearance. J. Mater. Sci. Technol..

[32] Baghirov H. (2025). Mechanisms of receptor-mediated transcytosis at the blood-brain barrier. J. Contr. Release.

[33] Gu Y., Zhang Q., Huang H., Ho K.H.W., Zhang Y., Yi C., Zheng Y., Chang R.C.C., Wang E.S., Yang M. (2024). Tau-targeting multifunctional nanocomposite based on tannic acid-metal for near-infrared fluorescence/magnetic resonance bimodal imaging-guided combinational therapy in Alzheimer's disease. Theranostics.

[34] Jia W., Tian H., Jiang J., Zhou L., Li L., Luo M., Ding N., Nice E.C., Huang C., Zhang H. (2023). Brain‐targeted HFn‐Cu‐REGO nanoplatform for site‐specific delivery and manipulation of autophagy and cuproptosis in glioblastoma. Small.

[35] Fan S., Zheng Y., Liu X., Fang W., Chen X., Liao W., Jing X., Lei M., Tao E., Ma Q., Zhang X., Guo R., Liu J. (2018). Curcumin-loaded PLGA-PEG nanoparticles conjugated with B6 peptide for potential use in Alzheimer's disease. Drug Deliv..

[36] Guo Q., Xu S., Yang P., Wang P., Lu S., Sheng D., Qian K., Cao J., Lu W., Zhang Q. (2020). A dual-ligand fusion peptide improves the brain-neuron targeting of nanocarriers in Alzheimer's disease mice. J. Contr. Release.

[37] Su Y., Huang Y., Kou Q., Lu L., Jiang H., Li X., Gui R., Huang R., Huang X., Ma J., Li J., Nie X. (2023). Study on the role of an erythrocyte membrane‐coated nanotheranostic system in targeted immune regulation of Alzheimer's disease. Adv. Sci..

[38] Zhang L., Liu X., Liu D., Yu X., Zhang L., Zhu J., Lu S., Liu R. (2020). A conditionally releasable “Do not Eat Me” CD47 signal facilitates microglia‐targeted drug delivery for the treatment of Alzheimer's disease. Adv. Funct. Mater..

[39] Jiang L., Zhu Y., Luan P., Xu J., Ru G., Fu J.-G., Sang N., Xiong Y., He Y., Lin G.-Q., Wang J., Zhang J., Li R. (2021). Bacteria*-*anchoring hybrid liposome capable of absorbing multiple toxins for antivirulence therapy of Escherichia coli infection. ACS Nano.

