# Supplementary material for: Multi-functional bionic nanoparticles for precise neuronal targeting and synergistic therapy in Alzheimer's disease
Source: Mater Today Bio. 2026 Aug 28;40:103617. doi: 10.1016/j.mtbio.2026.103617 (PMC13560336; doi:10.1016/j.mtbio.2026.103617)
Supplement: Supplementary file 1 [file mmc1.docx]

**Supporting Information**

**Multi-functional Bionic Nanoparticles for Precise Neuronal Targeting and Synergistic Therapy in** **Alzheimer's Disease**

Yue Na^1^, Chi Liu^2^, Xinming Bi^2^, Shumeng Liu^3^, Yue Xing^3^, Bohan Zhang^3^, Tongfeng Liu^2,^ *, Ning Zhang^3,^ *, Fang Geng^1,2,^ *

^1^College of Life Science and Technology, Harbin Normal University, Harbin, Heilongjiang, 150025, PR China

^2^Key Laboratory of Photochemistry Biomaterials & Energy Storage Materials of Heilongjiang Province, College of Chemistry & Chemical Engineering, Harbin Normal University, Harbin, Heilongjiang, 150025, PR China

^3^College of Pharmacy, Heilongjiang University of Chinese Medicine, Harbin, Heilongjiang 150004, PR China

*: Corresponding author. E-mail: Tongfeng Liu, priscillaliu95@163.com; Ning Zhang, zhangning0454@163.com; Fang Geng, f.geng@hrbnu.edu.cn

**This Supporting information contains:**

- Supporting Methods
- Supporting Tables S1 to S4
- Supporting Figures S1 to S27
- Supporting References

**Supporting Methods**

**Characterization**

The encapsulation efficiency (EE) and drug loading (DL) capacity of Gen and Don in the formulation were determined by high-performance liquid chromatography (HPLC) (Shimadzu, Japan). HPLC analysis was performed using an Agela C18 column (5.0 μm, 200 mm × 24.6 mm). The mobile phase for Gen consisted of water (A) and methanol (B), while the mobile phase for Don consisted of water (A) and methanol (B) containing 0.5% glacial acetic acid and 1% triethylamine. The injection volume was 20 μL.

Sodium dodecyl sulfate polyacrylamide gel electrophoresis (SDS-PAGE) was used to examine proteins located on the erythrocyte membrane surface, after which the gels were stained with Coomassie Brilliant Blue [1]. Protein concentrations of both the red blood cell membranes and the different biomimetic NPs were measured using a bicinchoninic acid (BCA) assay kit.

The stability of the prepared biomimetic NPs was analyzed by measuring their particle size over 72 hours while stored in 1× PBS or PBS containing 10% fetal bovine serum (FBS) at 37°C [2].

***In vitro* release profile**

To investigate the drug release kinetics, 1 mL of the Gen- and Don-loaded formulation was sealed inside a dialysis membrane with a 10 kDa molecular weight cutoff. This bag was then incubated in 30 mL of phosphate-buffered saline (PBS, 0.1 M, pH 7.4) under gentle agitation. At designated time points, 500 μL aliquots were taken from the external release medium and an equal volume of fresh PBS was immediately replenished to maintain a constant total volume. The concentrations of both Gen and Don in the withdrawn samples were subsequently determined using the HPLC method described previously.

**Combination index analysis**

Based on the single-drug activity data, BTRN nanoparticles were each formulated with a drug input of 2 mg (Gen or Don) and then mixed to yield a series of nano‑combinations at Gen: Don molar ratios of 1:1, 2:1, 3:1, 5:1, 1:2, 1:3 and 1:5. The protective effects of the resulting nano‑combinations against Aβ_25-35_‑induced cytotoxicity were evaluated in HT22 cells using the MTT assay as described above. Following viability determination, combination indices (CI) were calculated for each ratio employing CompuSyn software to assess the nature of drug interactions.

**Safety evaluation**

*In vivo safety evaluation*

Healthy mice were administered via tail vein injection with control group and BTRN group (5 mg/kg). Following euthanasia on day 14, major organs (spleen, liver, kidney, lung) were harvested. These tissues were processed and sectioned for slide mounting, followed by hematoxylin and eosin (H&E) staining to permit detailed histological examination.

*In vitro safety evaluation*

HT22, hCMEC/D3, and U-118MG cells were seeded into 96-well plates and cultured for 24 hours at 37 °C under 5% CO_2_. Subsequently, the cells were co-incubated with different concentrations of the nano-formulations for 24 hours under the same conditions. After incubation, cell viability was assessed using the MTT assay.

*Serum biochemistry and complete blood count analysis*

Blood samples were collected from the retro-orbital venous plexus of mice under light isoflurane anesthesia and divided into two portions. For serum biochemistry, whole blood was allowed to clot at room temperature for 30 min, and serum was separated by centrifugation at 3000 × g for 10 min at 4 °C. A standard panel of ten serum biochemical parameters—alanine aminotransferase (ALT), aspartate aminotransferase (AST), alkaline phosphatase (ALP), total protein (TP), albumin (ALB), total bilirubin (TBIL), blood urea nitrogen (BUN), creatinine (CREA), glucose (GLU), and total cholesterol (CHO)—was measured using an automated biochemistry analyzer. For complete blood count (CBC), whole blood was collected into EDTA-coated tubes and analyzed on an automated hematology analyzer to determine white blood cell (WBC) count, red blood cell (RBC) count, hemoglobin (HGB) concentration, hematocrit (HCT), mean corpuscular volume (MCV), mean corpuscular hemoglobin (MCH), mean corpuscular hemoglobin concentration (MCHC), and platelet (PLT) count. All assays were performed according to the manufacturers’ instructions.

**Pharmacokinetic Profiles**

Male ICR mice (6-8 weeks, 20-22 g) were randomly divided into four groups (n = 5 per group) and received a single intravenous injection via the tail vein of free Gen+Don, BTRN (Gen+Don) at a dose of 10 mg/kg. Blood samples (approximately 100 μL) were collected from the retro-orbital venous plexus into heparinized tubes at the following time points: 0.083 (5 min), 0.25, 0.5, 1, 2, 4, 8, 12, and 24 h post-administration. Plasma was separated by centrifugation at 3,000 × g for 10 min at 4 °C and stored at -80 °C until analysis. The concentrations of Gen and Don in plasma were simultaneously determined by a validated liquid chromatography-tandem mass spectrometry (LC-MS) method. Pharmacokinetic parameters, including the distribution half-life (t_1/2α_), terminal elimination half-life (t_1/2β_), total body clearance (CLz), area under the plasma concentration–time curve (AUC_0-t_), and mean residence time (MRT_0-t_), were calculated by non-compartmental analysis using DAS 2.0 software (Chinese Pharmacological Society).

**Cellular uptake**

After a 24-hour culture period (37℃, 5% CO_2_) in 6-well plates, HT22, hCMEC/D3, and U-118MG cells were used for qualitative assessment. The cells were incubated with FITC-loaded formulations (50 μg/mL) for 2 hours. Subsequently, they were fixed using 4% paraformaldehyde, counterstained with DAPI, and visualized under a fluorescence microscope (Olympus, Japan).

**Immune escape**

The immune evasion capability was evaluated by observing the phagocytosis of biomimetic nanocarriers by RAW 264.7 cells. Following a 24-hour culture period in 12-well plates under standard conditions (37℃, 5% CO_2_), RAW 264.7 cells were treated with different nanocarrier groups (50 μg/mL). After a 2-hour incubation, the cells were fixed with 4% paraformaldehyde for 15 minutes and subsequently stained with DAPI for 5 minutes to visualize nuclei. Cellular fluorescence was ultimately examined under a fluorescence microscope (Olympus, Japan).

***In vitro* BBB penetration ability evaluation**

*Cell co-culture BBB model*

The lower chamber of the Transwell insert (surface area 1.13 cm², pore size 0.4 μm) was first coated with a 2% gelatin solution. U-118MG cells were seeded in the lower chamber. After 24 hours of culture, hCMEC/D3 cells were seeded in the upper chamber of the insert and co-cultured for another 24 hours. Subsequently, HT22 cells were seeded into a 12-well plate, and the Transwell insert was placed into the well for an additional 24 hours of co-culture. The transepithelial electrical resistance (TEER) was measured using a Millicell ERS meter (Millipore Co., Bedford, MA, USA). Drug treatment was initiated when the TEER value exceeded 180 Ω·cm².

*BBB permeability assay*

One milliliter of FITC-loaded nanocarriers was added to the upper chamber of the Transwell insert. Samples (200 μL) were collected from the lower chamber at 0.5, 1, 2, 4, 6, and 12 hours, and the volume in the lower chamber was replenished with an equal amount of PBS. The fluorescence intensity of the nanocarriers before addition and the fluorescence intensity of the samples collected from the lower chamber were measured using a fluorescence spectrophotometer (PerkinElmer, USA).

**Small animal *in vivo* imaging**

The near-infrared dye DiR was used as a fluorescent probe to evaluate the brain-targeting ability of the biomimetic nanodelivery system. Various groups of DiR-loaded nano-formulations were administered to C57BL/6J mice via tail vein injection. The IVIS Lumina III system (Caliper Life Sciences, USA) was used for *in vivo* imaging of mice. Four hours after injection, the heart, liver, spleen, lungs, kidneys, and brain were rapidly collected to assess tissue distribution [3].

***In vivo* evaluation of central neuron targeting**

Following the intravenous administration of FITC-labeled nano-formulations (2 mg FITC/kg) via the tail vein to C57BL/6J mice, the animals were perfused with saline and 4% paraformaldehyde (PFA) at the 1-hour time point. The harvested brains were then post-fixed in 4% formaldehyde for 48 hours, cryoprotected in sucrose solution, and embedded in OCT compound for frozen sectioning. Tissue sections were blocked at 37℃ for 1 hour prior to incubation with a primary anti-NeuN antibody and the corresponding Alexa Fluor® 594-conjugated secondary antibody. Finally, nuclei were counterstained with a DAPI-containing mounting medium, and images were acquired using confocal microscope.

**Microdialysis coupled with Liquid Chromatography-Mass Spectrometry (LC-MS)**

Brain Microdialysate Collection: C57BL/6J mice were briefly anesthetized using isoflurane delivered via a gas anesthesia system. A microdialysis probe was implanted and positioned in the hippocampal tissue. After surgery, the mice were allowed to recover for 3-5 days. The pre-activated probe was then inserted, and the microdialysis perfusion system was started, perfusing artificial cerebrospinal fluid at a flow rate of 1.0 μL/min. After a 30-minute equilibration period, the drug was administered via tail vein injection. Brain microdialysate was continuously collected for 6 hours. Gen and Don were quantified using a Waters ACQUITY H-Class PLUS liquid chromatography system coupled with an AB SCIEX QTRAP® 5500 triple quadrupole tandem mass spectrometer operating in negative ion Multiple Reaction Monitoring (MRM) mode.

Luteolin was used as the internal standard for the analysis of genistein. Chromatographic separation was achieved using an ACQUITY UPLC BEH C18 column (1.7 μm, 2.1 × 50 mm) maintained at 40℃, with an injection volume of 5 μL. The mobile phase consisted of 0.1% aqueous formic acid and acetonitrile, delivered at a constant flow rate of 0.3 mL/min. Detection was carried out by tandem mass spectrometry in multiple reaction monitoring (MRM) mode, tracking the transitions of m/z 269.0→133.0 for genistein and m/z 285.1→133.1 for luteolin. Key mass spectrometric parameters were set as follows: capillary voltage, -4.50 kV; cone voltage, 20 V; collision energy, 25 eV; dwell time, 100 ms; source temperature, 120℃; desolvation temperature, 400℃. Argon served as the collision gas at a pressure of approximately 2.6 × 10^-3^ mbar, while nitrogen was used as both the desolvation and cone gas at a flow rate of 600 L/h for each.

For the quantification of donepezil using tacrine as the internal standard, chromatographic separation was achieved on an ACQUITY UPLC BEH C18 column (1.7 μm, 2.1 × 50 mm) maintained at 40℃ with an injection volume of 5 μL. The mobile phase, consisting of 5 mM ammonium formate in water and acetonitrile, was delivered at a flow rate of 0.5 mL/min. Detection was performed in multiple reaction monitoring (MRM) mode by tracking the specific mass transitions: m/z 380.2 → 91.1 for donepezil and m/z 199.2 → 144.1 for tacrine. Key mass spectrometric parameters were set as follows: capillary voltage, 4 kV; cone voltage, 50 V; collision energy, 15 eV; dwell time, 100 ms. The ion source temperature was 120℃ and the desolvation temperature was 400℃. Argon served as the collision gas at approximately 2.6 × 10^-3^ mbar, while nitrogen was used as both the desolvation and cone gas at a flow rate of 600 L/h for each.

To accurately quantify acetylcholine (ACh), six-month-old male APP/PS1 transgenic mice and age-matched wild-type (WT) littermates were randomly divided into treatment groups. All formulations were administered via tail vein injection at 2 mg/kg every other day for 30 days; control and model groups received an equivalent volume of saline. On the day after the final dose, mice were briefly anesthetized with isoflurane and a microdialysis probe was stereotaxically implanted into the hippocampus. After a 24h recovery, the pre-activated probe was perfused with artificial cerebrospinal fluid containing 0.5 μM neostigmine at 1.5 μL/min. Following a 2h equilibration, three consecutive basal dialysate fractions were collected at 30min intervals. Ach was measured using the same LC-MS system operated in positive-ion MRM mode. A HILIC column and a mobile phase of 0.1% formic acid in acetonitrile/water were used, with the transitions m/z 146.1→87.0 (ACh) and m/z 155.2→87.0 (d9-ACh internal standard) monitored.

**Behavioral test**

Six-month-old APP/PS1 mice were randomly divided into 9 groups (6 mice per group). They were administered via tail vein injection every other day with normal saline (control group), free Gen and Don solution, or different biomimetic nanocarriers loaded with Gen and/or Don for a total of 30 days, at a Gen and Don concentration of 2 mg/kg.

Open Field Test: The mice were gently placed in the center of a brightly lit arena (40 × 40 × 40.5 cm) and allowed to explore freely for 10 minutes. Its behavior was recorded automatically by an overhead video camera and subsequently analyzed for locomotor activity using the EthoVision XT tracking software [4].

Morris water maze (MWM): After the open field test, the mice underwent MWM training and testing [5]. Prior to testing, the mice underwent a training regimen consisting of three trials per day for five consecutive days. On the sixth day, a probe trial was conducted in which the platform was removed. Each animal was released into the pool from a predetermined starting point and allowed to swim freely for 90 seconds. The number of crossings over the former platform location was recorded. Swimming trajectories and latencies were quantified using an automated video tracking system (Shanghai Jiliang Software Technology Co., Ltd., China).

**HE staining**

For the morphological assessment of hippocampal neurons, mice brain tissues were collected and fixed in 10% formaldehyde, then embedded in paraffin. Subsequently, serial sections were obtained at an 8 μm thickness, mounted on glass slides, and stained with H&E [6]. Finally, the stained sections were examined and imaged using a light microscope.

**Cell apoptosis assay**

Apoptosis in HT22 cells was assessed by TUNEL staining according to the manufacturer's protocol [7]. Briefly, cells were seeded in 12-well plates and, after adherence, treated with the designated stimuli for the specified periods. Following treatment, cells were fixed with 4% paraformaldehyde and permeabilized with 0.3% Triton X-100 in ice-cold PBS. The TUNEL reaction mixture was then applied, and the samples were incubated at 37℃ for 1 hour in the dark. Finally, nuclei were counterstained with DAPI for 5 minutes, and fluorescence imaging was performed using a microscope (Olympus, Japan).

**Neuroprotective assessment *in vitro***

To assess potential neuroprotective effects, an AD cell model was first established by treating HT22 cells with 20 μmol/L Aβ_25-35_. For the experiment, cells were seeded in 96-well plates and pretreated with the various nanocarrier formulations for 24 hours. Following this pretreatment, the medium was removed and replaced with fresh medium containing 20 μmol/L Aβ_25-35_ for a further 24-hour incubation. Finally, cell viability was quantified using a standard MTT assay [8].

**Western blotting**

Brain tissues were homogenized and lysed in T-PER buffer supplemented with phosphatase and protease inhibitors (Roche) [9]. Following lysis, protein concentrations were determined by BCA assay. For each sample, 20 μg of protein was resolved by SDS-PAGE and transferred onto a PVDF membrane. After transfer, the membrane was blocked with 5% non-fat milk for 1 hour and then probed overnight at 4℃ with primary antibodies diluted in 1% BSA. This was followed by incubation with appropriate HRP-conjugated secondary antibodies. Protein bands were finally visualized using an ECL-Plus detection reagent.

To investigate the role of NF-κB signaling in BTRN-mediated regulation of BACE1 and Aβ, HT22 cells were exposed to 20 μM Aβ_25-35_ for 24 h and then treated with BTRN nanoparticles in the presence or absence of NF-κB activator 1 (compound 32, 5 μM) for an additional 24 h. Cells were lysed in RIPA buffer containing protease and phosphatase inhibitors, and protein concentrations were measured by BCA assay. Subsequent SDS-PAGE separation, membrane transfer, blocking, antibody incubation, and ECL detection were performed following the same procedure described above for brain tissue lysates. Protein bands were quantified using ImageJ software.

The antibody dilutions are as follows: SIRT1 1:1000; NF-κB p65 1:1000; BACE1 1:1000; APP 1:1000; Aβ 1:1000; Caspase3/p17/p19 1:1000; Caspase9/p35/p10 1:500.

**Immunohistochemistry and immunofluorescence analyses of the brain**

For immunohistochemical/immunofluorescence staining, 5μm brain sections were first subjected to antigen retrieval by heating in 0.01 M citrate buffer (pH 6.0) for 30 minutes. After washing with PBST, the sections were blocked by incubation with 10% normal goat serum for 2 hours. Subsequently, they were incubated with primary antibodies at 4℃ overnight [10].

In the IHC protocol, endogenous peroxidase activity was first quenched by incubating the sections in 3% H_2_O_2_ for 15 minutes. Following washes with PBST, the sections were incubated with HRP-conjugated secondary antibodies for 2 hours. Color development was then performed using ImmPACT^TM^ DAB substrate. The sections were subsequently rinsed with water, counterstained with hematoxylin, and mounted with EUKITT® medium. Finally, whole-slide images were captured using a panoramic scanner under standardized settings.

For IF staining, after primary antibody incubation, fluorophore-conjugated secondary antibodies were applied. The sections were mounted with an anti-fade mounting medium containing DAPI and observed under a fluorescence microscope (APX100, Olympus).

For cellular immunofluorescence detection of cytochrome c, HT22 cells were grown on glass coverslips, exposed to 20 μM Aβ_25-35_ for 24 h, and then treated with BTRN nanoparticles for an additional 24 h. Cells were fixed with 4 % paraformaldehyde, permeabilized with 0.1 % Triton X‑100, blocked with 5 % BSA, and incubated with anti‑cytochrome c antibody (1:200) overnight at 4 °C, followed by an Alexa Fluor‑conjugated secondary antibody and DAPI counterstaining.

**Quantitative real-time-PCR Analysis**

Total RNA was isolated from hippocampal tissues using the RNeasy Mini Kit (Qiagen). Following quantification and normalization, the RNA was reverse-transcribed into cDNA with HiScript® II Q RT SuperMix (Vazyme). Quantitative PCR was then performed on an Applied Biosystems 7500 Real-Time PCR System using ChamQ^TM^ SYBR Master Mix (Vazyme) and gene-specific primers designed via NCBI Primer-BLAST. Relative gene expression levels were calculated using the comparative ΔΔCt method, with β-Actin serving as the internal reference gene [11].

**Supporting Tables**

Table S1 Quantification of XPS spectra.

|  | RNPs | BRN | TRN | BTRN |
| --- | --- | --- | --- | --- |
| C | 63.05 | 70.72 | 67.29 | 57.21 |
| O | 35.28 | 25.85 | 28.73 | 38.53 |
| **N** | **1.67** | **3.43** | **3.98** | **4.26** |

Table S2 Combination index (CI) calculation data.

| Formulation | Ratio | CI value |
| --- | --- | --- |
| BTRN (Gen: Don) | 1:1 | 0.237 ± 0.028 |
|  | 2:1 | 0.396 ± 0.063 |
|  | 3:1 | 0.515 ± 0.045 |
|  | 5:1 | 0.726 ± 0.093 |
|  | 1:2 | 1.627 ± 0.384 |
|  | 1:3 | 2.336 ± 1.542 |
|  | 1:5 | 6.872 ± 2.744 |

Table S3 Serum biochemical and complete blood cell count test results.

|  | Parameter | PBS (Mean ± SD) | BTRN (Mean ± SD) |
| --- | --- | --- | --- |
| Serum biochemical | Albumin (g/L) | 32.4 ± 1.8 | 28.8 ± 3.1 |
|  | Total Protein (g/L) | 63.2 ± 3.4 | 61.7 ± 4.6 |
|  | Globulin(g/L) | 31.5 ± 1.9 | 32.3 ± 2.7 |
|  | Albumin/Globulin Ratio (A/G Ratio) | 1.34 ± 0.11 | 0.94 ± 0.08 |
|  | Alanine Aminotransferase (ALT) (U/L) | 45 ± 17 | 69 ± 14 |
|  | Alkaline Phosphatase (ALP) (U/L) | 149 ± 15 | 139 ± 22 |
|  | Creatinine (μmol/L) | 12.3 ± 1.9 | 12.7 ± 1.3 |
|  | Blood Urea Nitrogen (BUN) (mmol/L) | 8.48 ± 1.35 | 7.94 ± 1.87 |
|  | BUN/Creatinine Ratio | 655.3 ± 36.2 | 631.7 ± 47.3 |
|  | Glucose (mmol/L) | 9.33 ± 1.26 | 9.18 ± 1.11 |
| CBC | WBC (10^9/L) | 7.34 ± 1.33 | 8.86 ± 1.37 |
|  | Gran# (10^9/L) | 1.7 ± 0.39 | 1.8 ± 0.31 |
|  | Lym# (10^9/L) | 4.97 ± 0.63 | 4.77 ± 0.65 |
|  | Mon# (10^9/L) | 1.80 ± 0.24 | 1.84 ± 0.29 |
|  | Gran% (%) | 23.5 ± 4.5 | 20.3 ± 3.2 |
|  | Lym% (%) | 59.5 ± 5.7 | 57.6 ± 6.1 |
|  | Mon% (%) | 29.1 ± 4.3 | 25.2 ± 4.9 |
|  | RBC (10^12/L) | 8.21 ± 0.59 | 8.16 ± 0.45 |
|  | HGB (g/L) | 169 ± 16 | 181 ± 12 |
|  | HCT (%) | 43.2 ± 4.2 | 46.8 ± 3.7 |
|  | MCV (fL) | 56.3 ± 2.9 | 53.6 ± 1.8 |
|  | MCH (pg) | 23.6 ± 2.3 | 22.4 ± 3.1 |
|  | MCHC (g/L) | 441 ± 23 | 416 ± 29 |
|  | RDW-CV (%) | 13.4 ± 0.7 | 14.9 ± 1.3 |
|  | RDW-SD (fL) | 26.9 ± 2.6 | 29.3 ± 2.4 |
|  | PLT (10^9/L) | 1822 ± 123 | 1796 ± 171 |
|  | MPV (fL) | 5.8 ± 0.9 | 5.1 ± 0.7 |
|  | PDW | 13.9 ± 2.1 | 14.1 ± 2.3 |
|  | PCT (%) | 1.22 ± 0.21 | 1.16 ± 0.18 |
|  | P-LCC (10^9/L) | 475 ± 39 | 468 ± 57 |
|  | P-LCR (%) | 26.6 ± 4.4 | 30.2 ± 3.5 |

Table S4 Pharmacokinetic parameters of Gen and Don following tail vein administration in mice.

| Parameter | Unit | Gen | BTRN(Gen) | Don | BTRN(Don) |
| --- | --- | --- | --- | --- | --- |
| t_1/2α_ | h | 0.232 ± 0.005 | 0.640 ± 0.032 | 0.283 ± 0.006 | 0.785 ± 0.069 |
| t_1/2β_ | h | 2.361 ± 0.072 | 9.887 ± 0.204 | 2.065 ± 0.032 | 11.532 ± 0.561 |
| CLz | L/h/kg | 7.593 ± 0.061 | 0.553 ± 0.002 | 6.832 ± 0.053 | 0.537 ± 0.013 |
| AUC_0-t_ | μg/mL·h | 0.264 ± 0.002 | 3.616 ±0. 012 | 0.293 ± 0.002 | 3.728 ± 0.086 |
| MRT_0-t_ | h | 0.502 ± 0.003 | 12.464 ± 0.260 | 0.648 ± 0.017 | 14.183 ± 0.602 |

Figure S1 ^1^H-NMR of B6 and DSPE-PEG_2000_-B6.

Figure S2 ^1^H-NMR of Tet1 and DSPE-PEG_2000_-Tet1.

Figure S3 XPS survey spectra of bio-nanoparticles from different groups.

Figure S4 UV-vis absorption spectra for bio-nanoparticles from different groups.

Figure S5 Representative WB images of CD47 expression.

Figure S6 Cell viability of Aβ_25-35_‑induced HT22 cells following treatment with nanoparticles prepared at different ratios. **p* < 0.05, ***p* < 0.01, ****p* < 0.001, compared with model group.

Figure S7 MTT cell viability of HT22 cells incubated for 24 h with different concentrations of free Gen+Don, NPs (Gen+Don), RNPs (Gen+Don), BTRN (Gen+Don), and empty BTRN (n = 5).

Figure S8 MTT cell viability of hCMEC/D3 cells incubated for 24 h with different concentrations of free Gen+Don, NPs (Gen+Don), RNPs (Gen+Don), BTRN (Gen+Don), and empty BTRN (n = 5).

Figure S9 MTT cell viability of U-118MG cells incubated for 24 h with different concentrations of free Gen+Don, NPs (Gen+Don), RNPs (Gen+Don), BTRN (Gen+Don), and empty BTRN (n = 5).

Figure S10 Representative histological staining of major organs from healthy mice treated with BTRN (scale bar = 50 μm).

Figure S11 Quantitative analysis of RAW264.7 cell uptake. **p* < 0.05, ***p* < 0.01, compared with NPs group.

Figure S12 Quantitative analysis of hCMEC/D3 cell uptake. **p* < 0.05, ****p* < 0.001, compared with NPs group.

Figure S13 Quantitative analysis of U-118MG cell uptake. **p* < 0.05, ****p* < 0.001, compared with NPs group.

Figure S14 Fluorescence microscopy images of HT22 cell uptake of fluorescently labeled NPs (scale bar = 200 μm).

Figure S15 Quantitative analysis of HT22 cell uptake. **p* < 0.05, ***p* < 0.01, ****p* < 0.001, compared with NPs group.

Figure S16 Pearson's coefficient of neuronal colocalization (n = 3). **p* < 0.05, ***p* < 0.01, ****p* < 0.001, compared with NPs group.

Figure S17 Representative immunohistochemical quantification results showed the Aβ levels in the CA1 regions of the hippocampus in APP/PS1 mice. #*p* < 0.05, compared with the control group. **p* < 0.05, compared with model group.

Figure S18 Representative immunohistochemical quantification results showed the Aβ levels in the CA3 regions of the hippocampus in APP/PS1 mice. ##*p* < 0.01, compared with the control group. **p* < 0.05, ***p* < 0.01, compared with model group.

Figure S19 Therapeutic effects of various groups of biological NPs on HT22 cells induced by Aβ_25-35_. **p* < 0.05, ***p* < 0.01, ****p* < 0.001, compared with model group (n=5).

Figure S20 Representative WB images of BACE1 and Aβ protein levels in Aβ_25-35_‑induced HT22 cells following specific treatment with NF‑κB agonist 1.

Figure S21 Quantification of BACE1 protein levels in Aβ_25-35_‑induced HT22 cells following specific treatment with NF‑κB agonist 1. ****p* < 0.001, compared with control group.

Figure S22 Quantification of Aβ protein levels in Aβ_25-35_‑induced HT22 cells following specific treatment with NF‑κB agonist 1. ****p* < 0.001, compared with control group.

Figure S23 Representative immunofluorescence quantification results of CHAT. ###*p* < 0.001 compared with the control group. **p* < 0.05, ***p* < 0.01, ****p* < 0.001, compared with model group.

Figure S24 Representative immunofluorescence quantification results of α7-nAChR. ###*p* < 0.001, compared with the control group. **p* < 0.05, ***p* < 0.01, compared with model group.

Figure S25 HE staining in hippocampal CA1 regions of APP/PS1 (scale bar: 50 μm).

Figure S26 HE staining in hippocampal dentate gyrus regions of APP/PS1 (scale bar: 50 μm).

Figure S27 Immunofluorescence images of Cyt c in HT22 cells (scale bar: 50 μm).

**Reference**

[1] J. Xie, Q. Shen, K. Huang, T. Zheng, L. Cheng, Z. Zhang, Y. Yu, G. Liao, X. Wang, C. Li, Oriented assembly of cell-mimicking nanoparticles via a molecular affinity strategy for targeted drug delivery, ACS Nano 13 (2019) 5268-5277. https://doi.org/10.1021/acsnano.8b09681.

[2] W. Hao, Y. Cui, Y. Fan, M. Chen, G. Yang, Y. Wang, M. Yang, Z. Li, W. Gong, Y. Yang, C. Gao, Hybrid membrane-coated nanosuspensions for multi-modal anti-glioma therapy via drug and antigen delivery, J. Nanobiotechnol. 19 (2021) 378. https://doi.org/10.1186/s12951-021-01110-0.

[3] F. Yang, D. Zhao, M. Cheng, Y. Liu, Z. Chen, J. Chang, Y. Dou, mTOR-mediated immunometabolic reprogramming nanomodulators enable sensitive switching of energy deprivation-induced microglial polarization for Alzheimer’s disease management, ACS Nano 17 (2023) 15724-15741. https://doi.org/10.1021/acsnano.3c03232.

[4] R. Lin, L. Jin, Y. Xue, Z. Zhang, H. Huang, D. Chen, Q. Liu, Z. Mao, Z. Wu, Q. Tao, Hybrid membrane‐coated nanoparticles for precise targeting and synergistic therapy in Alzheimer’s disease, Adv. Sci. 11 (2024). https://doi.org/10.1002/advs.202306675.

[5] L. Wang, W. Zhou, H. Yang, F. Liu, Y. Kong, W. Wang, H. Zhao, W. Ma, Y. Sang, F. Yi, H. Liu, C. Liu, A. Hao, J. Qiu, Calcium folate nanoparticles as dual‐functional neural inducing factors to promote the differentiation of neural stem cells into cholinergic neurons, Adv. Funct. Mater. 33 (2023) 2208835. https://doi.org/10.1002/adfm.202208835.

[6] W. Qian, D. Liu, J. Liu, M. Liu, Q. Ji, B. Zhang, Z. Yang, Y. Cheng, S. Zhou, The mitochondria‐targeted micelle inhibits Alzheimer’s disease progression by alleviating neuronal mitochondrial dysfunction and neuroinflammation, Small 21 (2025) 2408581. https://doi.org/10.1002/smll.202408581.

[7] Z. He, X. Li, Z. Wang, Y. Cao, S. Han, N. Li, J. Cai, S. Cheng, Q. Liu, Protective effects of luteolin against amyloid beta-induced oxidative stress and mitochondrial impairments through peroxisome proliferator-activated receptor γ-dependent mechanism in Alzheimer’s disease, Redox Biol. 66 (2023) 102848. https://doi.org/10.1016/j.redox.2023.102848.

[8] J. Gu, C. Yan, S. Yin, H. Wu, C. Liu, A. Xue, X. Lei, N. Zhang, F. Geng, Erythrocyte membrane-coated nanocarriers modified by TGN for Alzheimer’s disease, J. Control. Release 366 (2024) 448-459. https://doi.org/10.1016/j.jconrel.2023.12.030.

[9] J. Huang, F. Wu, W. Cao, Y. Chen, Q. Yao, P. Cen, J. Wang, L. Hong, X. Zhang, R. Zhou, C. Jin, M. Tian, H. Zhang, Y. Zhong, Ultrasmall iron-gallic acid coordination polymer nanoparticles for scavenging ROS and suppressing inflammation in tauopathy-induced Alzheimer’s disease, Biomaterials 317 (2025) 123042. https://doi.org/10.1016/j.biomaterials.2024.123042.

[10] P. Liu, T. Zhang, Q. Chen, C. Li, Y. Chu, Q. Guo, Y. Zhang, W. Zhou, H. Chen, Z. Zhou, Y. Wang, Z. Zhao, Y. Luo, X. Li, H. Song, B. Su, C. Li, T. Sun, C. Jiang, Biomimetic dendrimer–peptide conjugates for early multi‐target therapy of Alzheimer’s disease by inflammatory microenvironment modulation, Adv. Mater. 33 (2021) 2100746. https://doi.org/10.1002/adma.202100746.

[11] L. Tang, J. Wang, J. Zhu, Y. Liang, Down‐regulated SHARPIN may accelerate the development of atopic dermatitis through activating interleukin‐33/ST2 signaling, Exp. Dermatol. 27 (2018) 1328. https://doi.org/10.1111/exd.13784.
